# Author Correction: Discovery of 42 genome-wide significant loci associated with dyslexia

**DOI:** 10.1038/s41588-023-01336-8

**Published:** 2023-02-23

**Authors:** Catherine Doust, Pierre Fontanillas, Else Eising, Scott D. Gordon, Zhengjun Wang, Gökberk Alagöz, Barbara Molz, 23andMe Research Team, Stella Aslibekyan, Adam Auton, Elizabeth Babalola, Robert K. Bell, Jessica Bielenberg, Katarzyna Bryc, Emily Bullis, Daniella Coker, Gabriel Cuellar Partida, Devika Dhamija, Sayantan Das, Sarah L. Elson, Teresa Filshtein, Kipper Fletez-Brant, Will Freyman, Pooja M. Gandhi, Karl Heilbron, Barry Hicks, David A. Hinds, Ethan M. Jewett, Yunxuan Jiang, Katelyn Kukar, Keng-Han Lin, Maya Lowe, Jey McCreight, Matthew H. McIntyre, Steven J. Micheletti, Meghan E. Moreno, Joanna L. Mountain, Priyanka Nandakumar, Elizabeth S. Noblin, Jared O’Connell, Aaron A. Petrakovitz, G. David Poznik, Morgan Schumacher, Anjali J. Shastri, Janie F. Shelton, Jingchunzi Shi, Suyash Shringarpure, Vinh Tran, Joyce Y. Tung, Xin Wang, Wei Wang, Catherine H. Weldon, Peter Wilton, Alejandro Hernandez, Corinna Wong, Christophe Toukam Tchakouté, Quantitative Trait Working Group of the GenLang Consortium, Filippo Abbondanza, Andrea G. Allegrini, Till F. M. Andlauer, Cathy L. Barr, Manon Bernard, Kirsten Blokland, Milene Bonte, Dorret I. Boomsma, Thomas Bourgeron, Daniel Brandeis, Manuel Carreiras, Fabiola Ceroni, Valéria Csépe, Philip S. Dale, Peter F. de Jong, Jean Francois Démonet, Eveline L. de Zeeuw, Yu Feng, Marie-Christine J. Franken, Margot Gerritse, Alessandro Gialluisi, Sharon L. Guger, Marianna E. Hayiou-Thomas, Juan Hernández-Cabrera, Jouke-Jan Hottenga, Charles Hulme, Philip R. Jansen, Juha Kere, Elizabeth N. Kerr, Tanner Koomar, Karin Landerl, Gabriel T. Leonard, Zhijie Liao, Maureen W. Lovett, Heikki Lyytinen, Angela Martinelli, Urs Maurer, Jacob J. Michaelson, Nazanin Mirza-Schreiber, Kristina Moll, Angela T. Morgan, Bertram Müller-Myhsok, Dianne F. Newbury, Markus M. Nöthen, Tomas Paus, Zdenka Pausova, Craig E. Pennell, Robert J. Plomin, Kaitlyn M. Price, Franck Ramus, Sheena Reilly, Louis Richer, Kaili Rimfeld, Gerd Schulte-Körne, Chin Yang Shapland, Nuala H. Simpson, Margaret J. Snowling, John F. Stein, Lisa J. Strug, Henning Tiemeier, J. Bruce Tomblin, Dongnhu T. Truong, Elsje van Bergen, Marc P. van der Schroeff, Marjolein Van Donkelaar, Ellen Verhoef, Carol A. Wang, Kate E. Watkins, Andrew J. O. Whitehouse, Karen G. Wigg, Margaret Wilkinson, Gu Zhu, Beate St Pourcain, Clyde Francks, Riccardo E. Marioni, Jingjing Zhao, Silvia Paracchini, Joel B. Talcott, Anthony P. Monaco, John F. Stein, Jeffrey R. Gruen, Richard K. Olson, Erik G. Willcutt, John C. DeFries, Bruce F. Pennington, Shelley D. Smith, Margaret J. Wright, Nicholas G. Martin, Adam Auton, Timothy C. Bates, Simon E. Fisher, Michelle Luciano

**Affiliations:** 1 Department of Psychology, University of Edinburgh, Edinburgh, UK; 2 23andMe, Inc., Sunnyvale, CA USA; 3 Language and Genetics Department, Max Planck Institute for Psycholinguistics, Nijmegen, the Netherlands; 4 Genetic Epidemiology Laboratory, QIMR Berghofer Medical Research Institute, Brisbane, Queensland Australia; 5 School of Psychology, Shaanxi Normal University and Shaanxi Key Research Center of Child Mental and Behavioral Health, Xi’an, China; 6 Donders Institute for Brain, Cognition and Behaviour, Radboud University, Nijmegen, the Netherlands; 7 MRC Integrative Epidemiology Unit, University of Bristol, Bristol, UK; 8 Centre for Genomic and Experimental Medicine, Institute of Genetics and Cancer, University of Edinburgh, Edinburgh, UK; 9 School of Medicine, University of St Andrews, St Andrews, UK; 10 Institute of Health and Neurodevelopment, Aston University, Birmingham, UK; 11 Office of the President, Tufts University, Medford, MA USA; 12 Department of Physiology, Anatomy and Genetics, Oxford University, Oxford, UK; 13 Departments of Pediatrics and Genetics, Yale Medical School, New Haven, CT USA; 14 Department of Psychology and Neuroscience, University of Colorado, Boulder, CO USA; 15 Institute for Behavioral Genetics, University of Colorado, Boulder, CO USA; 16 Department of Psychology, University of Denver, Denver, CO USA; 17 Department of Neurological Sciences, College of Medicine, University of Nebraska Medical Center, Omaha, NE USA; 18 Queensland Brain Institute, University of Queensland, Brisbane, Queensland Australia; 19 Social, Genetic and Developmental Psychiatry Centre, Institute of Psychiatry, Psychology and Neuroscience, King’s College London, London, UK; 20 Translational Research in Psychiatry, Max Planck Institute of Psychiatry, Munich, Germany; 21 Department of Neurology, Klinikum rechts der Isar, School of Medicine,Technical University of Munich, Munich, Germany; 22 Division of Experimental and Translational Neuroscience, Krembil Research Institute, University Health Network, Toronto, Ontario Canada; 23 Program in Neuroscience and Mental Health, Hospital for Sick Children, Toronto, Ontario Canada; 24 Department of Physiology, University of Toronto, Toronto, Ontario Canada; 25 Departments of Physiology and Nutritional Sciences, Hospital for Sick Children, Toronto, Ontario Canada; 26 Department of Cognitive Neuroscience and Maastricht Brain Imaging Center, Faculty of Psychology and Neuroscience, Maastricht University, Maastricht, the Netherlands; 27 Netherlands Twin Register, Amsterdam, the Netherlands; 28 Department of Biological Psychology, Vrije Universiteit Amsterdam, Amsterdam, the Netherlands; 29 Amsterdam Reproduction and Development (AR&D) Research Institute, Amsterdam, the Netherlands; 30 Human Genetics and Cognitive Functions Unit, Institut Pasteur, Paris, France; 31 CNRS UMR 3571, Université de Paris, Paris, France; 32 Department of Child and Adolescent Psychiatry and Psychotherapy, Psychiatric Hospital, University of Zurich, Zurich, Switzerland; 33 Zurich Center for Integrative Human Physiology (ZIHP), University of Zurich and ETH Zurich, Zurich, Switzerland; 34 Neuroscience Center Zurich, University of Zurich and ETH Zurich, Zurich, Switzerland; 35 Department of Child and Adolescent Psychiatry and Psychotherapy, Central Institute of Mental Health, Medical Faculty Mannheim, Heidelberg University, Mannheim, Germany; 36 Basque Center on Cognition, Brain and Language (BCBL), Donostia-San Sebastian, Spain; 37 Ikerbasque, Basque Foundation for Science, Bilbao, Spain; 38 Lengua Vasca y Comunicación, University of the Basque Country (UPV/EHU), Bilbao, Spain; 39 Department of Pharmacy and Biotechnology, University of Bologna, Bologna, Italy; 40 Faculty of Health and Life Sciences, Oxford Brookes University, Oxford, UK; 41 Brain Imaging Centre, Research Centre for Natural Sciences, Budapest, Hungary; 42 Multilingualism Doctoral School, Faculty of Modern Philology and Social Sciences, University of Pannonia, Veszprém, Hungary; 43 Department of Speech and Hearing Sciences, University of New Mexico, Albuquerque, NM USA; 44 Department of Child Development and Education, University of Amsterdam, Amsterdam, the Netherlands; 45 Leenaards Memory Centre, Department of Clinical Neurosciences Lausanne University Hospital (CHUV), University of Lausanne, Lausanne, Switzerland; 46 Genetics and Development Division, Krembil Research Institute, University Health Network, Toronto, Ontario Canada; 47 Department of Otorhinolaryngology, Erasmus University Medical Centre, Rotterdam, the Netherlands; 48 Department of Epidemiology and Prevention, IRCCS Istituto Neurologico Mediterraneo Neuromed, Pozzilli, Italy; 49 Department of Psychology, Hospital for Sick Children, Toronto, Ontario Canada; 50 Department of Psychology, University of York, York, UK; 51 Departamento de Psicología Clínica Psicobiología y Metodología, Universidad de La Laguna, Santa Cruz de Tenerife, Spain; 52 Department of Education, University of Oxford, Oxford, UK; 53 Department of Child and Adolescent Psychiatry/Psychology, Erasmus University Medical Center, Rotterdam, the Netherlands; 54 Department of Complex Trait Genetics, Center for Neurogenomics and Cognitive Research, Amsterdam Neuroscience, VU University, Amsterdam, the Netherlands; 55 Department of Human Genetics, VU Medical Center, Amsterdam UMC, Amsterdam, the Netherlands; 56 Department of Biosciences and Nutrition, Karolinska Institutet, Stockholm, Sweden; 57 Stem Cells and Metabolism Research Program, University of Helsinki, and Folkhälsan Research Center, Helsinki, Finland; 58 Department of Neurology, Hospital for Sick Children, Toronto, Ontario Canada; 59 Department of Paediatrics, The University of Toronto, Toronto, Ontario Canada; 60 Department of Psychiatry, University of Iowa, Iowa City, IA USA; 61 Institute of Psychology, University of Graz, Graz, Austria; 62 BioTechMed-Graz, Graz, Austria; 63 Cognitive Neuroscience Neurology and Neurosurgery, Montreal, Quebec Canada; 64 Department of Psychology, University of Toronto, Toronto, Ontario Canada; 65 Department of Psychology, University of Jyväskylä, Jyväskylä, Finland; 66 Department of Psychology, The Chinese University of Hong Kong, Hong Kong, China; 67 Institute of Neurogenomics, Helmholtz Zentrum München, Munich, Germany; 68 Department of Child and Adolescent Psychiatry, Psychosomatics, and Psychotherapy, LMU University Hospital Munich, Munich, Germany; 69 Speech and Language, Murdoch Children’s Research Institute, Melbourne, Victoria Australia; 70 Department of Audiology and Speech Pathology, University of Melbourne, Melbourne, Victoria Australia; 71 Speech Pathology Department, Royal Children’s Hospital, Melbourne, Victoria Australia; 72 Department of Health Science, University of Liverpool, Liverpool, UK; 73 Institute of Human Genetics, University Hospital of Bonn, Bonn, Germany; 74 Department of Psychiatry, University of Toronto, Toronto, Ontario Canada; 75 Departments of Psychiatry and Neuroscience and Centre Hospitalier Universitaire Sainte Justine, University of Montreal, Montreal, Quebec Canada; 76 Department of Psychology, University of Toronto, Toronto, Ontario Canada; 77 Hospital for Sick Children, Toronto, Ontario Canada; 78 School of Medicine and Public Health, College of Health, Medicine and Wellbeing, University of Newcastle, Newcastle, New South Wales Australia; 79 Mothers and Babies Research Centre, Hunter Medical Research Institute, Newcastle, New South Wales Australia; 80 Maternity and Gynaecology, John Hunter Hospital, Newcastle, New South Wales Australia; 81 Laboratoire de Sciences Cognitives et Psycholinguistique, Ecole Normale Supérieure, PSL University, EHESS, CNRS, Paris, France; 82 Menzies Health Institute Queensland, Griffith University, Gold Coast, Queensland Australia; 83 Department of Health Sciences, Université du Québec à Chicoutimi, Chicoutimi, Quebec Canada; 84 Population Health Sciences, University of Bristol, Bristol, UK; 85 Department of Experimental Psychology, University of Oxford, Oxford, UK; 86 St John’s College, University of Oxford, Oxford, UK; 87 Department of Physiology, Anatomy and Genetics, Oxford University, Oxford, UK; 88 Departments of Statistical Sciences and Computer Science and Division of Biostatistics, University of Toronto, Toronto, Ontario Canada; 89 Program in Genetics and Genome Biology and The Centre for Applied Genomics, Hospital For Sick Children, Toronto, Ontario Canada; 90 Harvard T.H. Chan School of Public Health, Boston, MA USA; 91 Communication Sciences and Disorders, University of Iowa, Iowa City, IA USA; 92 Research Institute LEARN!, Vrije Universiteit Amsterdam, Amsterdam, the Netherlands; 93 Department of Otolaryngology, Head and Neck Surgery, Erasmus MC, Rotterdam, the Netherlands; 94 Generation R Study Group, Erasmus MC, Rotterdam, the Netherlands; 95 Telethon Kids Institute, The University of Western Australia, Perth, Western Australia Australia

**Keywords:** Psychiatric disorders, Genome-wide association studies, Psychology

Correction to: *Nature Genetics* 10.1038/s41588-022-01192-y. Published online 20 October 2022.

In the version of this article originally published, a paragraph was omitted in the Methods section, reading “**Genomic control**. Top SNPs are reported from the more conservative GWAS results adjusted for genomic control (Fig. 1, Extended Data Figs. 1–4, and Supplementary Tables 1, 2, 9 and 10), whereas downstream analyses (including gene-set analysis, enrichment and heritability partitioning, genetic correlations, polygenic prediction, candidate gene replication) are based on GWAS results without genomic control.” The paragraph has now been included in the HTML and PDF versions of the article.

