# Supplementary material for: Discovery of 42 genome-wide significant loci associated with dyslexia
Source: Nat Genet. 2022 Oct 20;54(11):1621–9. doi: 10.1038/s41588-022-01192-y (PMC9649434; doi:10.1038/s41588-022-01192-y)
Supplement: Supplementary file 1 — Supplementary Note and Fig. 1. [file 41588_2022_1192_MOESM1_ESM.pdf]

nature genetics

Article

https://doi.org/10.1038/s41588-022-01192-y

Discovery of 42 genome-wide significant loci associated with dyslexia

In the format provided by the authors and unedited

SUPPLEMENTARY INFORMATION

Table of Contents

METHODS 5

23andMe Genotyping and imputation 5

Chinese Reading Study sample 6

Participants 6

Phenotypic measures 7

Genotype quality control, imputation, and analysis 7

Biological annotations 8

Partitioned heritability 10

REFERENCES 12

GENLANG QUANTITATIVE TRAIT CONSORTIUM ACKNOWLEDGEMENTS 19

FIGURES 26

Supplementary Figure 1.i. Regional association plot which includes information on the credible variant set and known genes in the region for the significant association between dyslexia and chr9p22.3 esv3619796 structural variant nearby rs3122702 26

Supplementary Figure 1.ii. Regional association plot which includes information on the credible variant set and known genes in the region for the significant association between dyslexia and chr3q22.3 rs13082684 27

Supplementary Figure 1.iii. Regional association plot which includes information on the credible variant set and known genes in the region for the significant association between dyslexia and chr20q13.13 rs11393101 28

Supplementary Figure 1.iv. Regional association plot which includes information on the credible variant set and known genes in the region for the significant association between dyslexia and chr17q12 rs34349354 29

Supplementary Figure 1.v. Regional association plot which includes information on the credible variant set and known genes in the region for the significant association between dyslexia and chr9q34.11 rs9696811 30

Supplementary Figure 1.vi. Regional association plot which includes information on the credible variant set and known genes in the region for the significant association between dyslexia and chr20q11.21 rs4911257 31

Supplementary Figure 1.vii. Regional association plot which includes information on the credible variant set and known genes in the region for the significant association between dyslexia and chr11q23.1 rs138127836 32

Supplementary Figure 1.viii. Regional association plot which includes information on the credible variant set and known genes in the region for the significant association between dyslexia and chr17q12 rs12453682 33

Supplementary Figure 1.ix. Regional association plot which includes information on the credible variant set and known genes in the region for the significant association between dyslexia and chr2q33.1 rs72916919 34

Supplementary Figure 1.x. Regional association plot which includes information on the credible variant set and known genes in the region for the significant association between dyslexia and chr11p14.1 rs676217 35

Supplementary Figure 1.xi. Regional association plot which includes information on the credible variant set and known genes in the region for the significant association between dyslexia and chr1p32.1 rs12737449 36

Supplementary Figure 1.xii. Regional association plot which includes information on the credible variant set and known genes in the region for the significant association between dyslexia and chr4q31.3 rs4696277 37

Supplementary Figure 1.xiii. Regional association plot which includes information on the credible variant set and known genes in the region for the significant association between dyslexia and chr12q24.12 rs7310615 38

Supplementary Figure 1.xiv. Regional association plot which includes information on the credible variant set and known genes in the region for the significant association between dyslexia and chr10q24.2 rs10786387 39

Supplementary Figure 1.xv. Regional association plot which includes information on the credible variant set and known genes in the region for the significant association between dyslexia and chr5q34 rs41012 40

Supplementary Figure 1.xvi. Regional association plot which includes information on the credible variant set and known genes in the region for the significant association between dyslexia and chr7q22.3 rs3839821 41

Supplementary Figure 1.xvii. Regional association plot which includes information on the credible variant set and known genes in the region for the significant association between dyslexia and chr3p12.1 rs10511073 42

Supplementary Figure 1.xviii. Regional association plot which includes information on the credible variant set and known genes in the region for the significant association between dyslexia and chr1q21.3 rs4845687 43

Supplementary Figure 1.xix. Regional association plot which includes information on the credible variant set and known genes in the region for the significant association between dyslexia and chr3p24.3 rs373178590 44

Supplementary Figure 1.xx. Regional association plot which includes information on the credible variant set and known genes in the region for the significant association between dyslexia and chr3p13 rs13097431 45

Supplementary Figure 1.xxi. Regional association plot which includes information on the credible variant set and known genes in the region for the significant association between dyslexia and chr2p22.1 rs906549 46

Supplementary Figure 1.xxii. Regional association plot which includes information on the credible variant set and known genes in the region for the significant association between dyslexia and chr1p13.3 rs2091329 47

Supplementary Figure 1.xxiii. Regional association plot which includes information on the credible variant set and known genes in the region for the significant association between dyslexia and chr2q22.3 rs497418 48

Supplementary Figure 1.xxiv. Regional association plot which includes information on the credible variant set and known genes in the region for the significant association between dyslexia and chr3p21.31 rs2624839 49

Supplementary Figure 1.xxv. Regional association plot which includes information on the credible variant set and known genes in the region for the significant association between dyslexia and chrXq27.3 rs5904158 50

Supplementary Figure 1.xxvi. Regional association plot which includes information on the credible variant set and known genes in the region for the significant association between dyslexia and chr10q24.33 rs34732054 51

Supplementary Figure 1.xxvii. Regional association plot which includes information on the credible variant set and known genes in the region for the significant association between dyslexia and chr3q26.33 rs7625418 52

Supplementary Figure 1.xxviii. Regional association plot which includes information on the credible variant set and known genes in the region for the significant association between dyslexia and chr2q33.1 rs6435017 53

Supplementary Figure 1.xxix. Regional association plot which includes information on the credible variant set and known genes in the region for the significant association between dyslexia and chr17q23.3 rs72841395 54

Supplementary Figure 1.xxx. Regional association plot which includes information on the credible variant set and known genes in the region for the significant association between dyslexia and chr13q12.13 rs375018025 55

Supplementary Figure 1.xxxi. Regional association plot which includes information on the credible variant set and known genes in the region for the significant association between dyslexia and chr14q32.2 rs35131341 56

Supplementary Figure 1.xxxii. Regional association plot which includes information on the credible variant set and known genes in the region for the significant association between dyslexia and chr5q35.1 rs59261790 57

Supplementary Figure 1.xxxiii. Regional association plot which includes information on the credible variant set and known genes in the region for the significant association between dyslexia and chr9p22.3 rs3122702 58

Supplementary Figure 1.xxxiv. Regional association plot which includes information on the credible variant set and known genes in the region for the significant association between dyslexia and chr2q12.1 rs367982014 59

Supplementary Figure 1.xxxv. Regional association plot which includes information on the credible variant set and known genes in the region for the significant association between dyslexia and chr12q24.31 rs4767921 60

Supplementary Figure 1.xxxvi. Regional association plot which includes information on the credible variant set and known genes in the region for the significant association between dyslexia and chr5q33.3 rs867009 61

Supplementary Figure 1.xxxvii. Regional association plot which includes information on the credible variant set and known genes in the region for the significant association between dyslexia and chr19q13.2 rs60963584 62

Supplementary Figure 1.xxxviii. Regional association plot which includes information on the credible variant set and known genes in the region for the significant association between dyslexia and chr7q11.22 rs77059784 63

Supplementary Figure 1.xxxix. Regional association plot which includes information on the credible variant set and known genes in the region for the significant association between dyslexia and chr2p23.2 rs1969131 64

Supplementary Figure 1.xl. Regional association plot which includes information on the credible variant set and known genes in the region for the significant association between dyslexia and chr7p14.1 rs62453457 65

Supplementary Figure 1.xli. Regional association plot which includes information on the credible variant set and known genes in the region for the significant association between dyslexia and chr6p22.3 rs2876430 66

Supplementary Figure 1.xlii. Regional association plot which includes information on the credible variant set and known genes in the region for the significant association between dyslexia and chr1q41 rs35570426 67

Supplementary Figure 1.xliii. Regional association plot which includes information on the credible variant set and known genes in the region for the significant association between dyslexia and chr7q11.22 rs3735260 68

METHODS

23andMe Genotyping and imputation

Samples were genotyped on one of five genotyping platforms. The V1 and V2 platforms were variants of the Illumina HumanHap550 + BeadChip, including about 25,000 custom SNPs selected by 23andMe, with a total of about 560,000 SNPs. The V3 platform was based on the Illumina OmniExpress + BeadChip, with custom content to improve the overlap with our V2 array, with a total of ~950,000 SNPs. The V4 platform is a fully custom array, including a lower redundancy subset of V2 and V3 SNPs with additional coverage of lower-frequency coding variation, and ~570,000 SNPs. The v5 platform, in current use, is an Illumina Infinium Global Screening Array (~640,000 SNPs) supplemented with ~50,000 SNPs of custom content. Samples that failed to reach 98.5% call rate were excluded from the study.

Individuals were only included if they had > 97% European ancestry, as determined through an analysis of local ancestry (see [1] for further details on the methodology used). Briefly, this analysis first partitions phased genomic data into short windows of ~100 SNPs. Within each window, a support vector machine is used to classify individual haplotypes into one of 31 reference populations. The support vector machine classifications are then fed into a Hidden Markov Model (HMM) that accounts for switch errors and incorrect assignments and gives probabilities for each reference population in each window. Finally, simulated admixed individuals are used to recalibrate the HMM probabilities so that the reported assignments are consistent with the simulated admixture proportions. The reference population data are derived from public data sets (the Human Genome Diversity Project, HapMap and 1000 Genomes) and from 23andMe research participants who have reported having four grandparents from the same country.

A maximal set of unrelated individuals was chosen for each analysis using a segmental identity-by-descent (IBD) estimation algorithm [2]. Individuals were defined as related if they shared more than 700 cM IBD, including regions where the two individuals share either one or both genomic segments identical-by-descent. This level of relatedness (roughly 20% of the genome) corresponds

approximately to the minimal expected sharing between first cousins in an outbred population. For the purposes of GWAS, if a case was found to be related to a control, the case was preferentially kept in the sample.

Participant genotype data were imputed against a single unified imputation reference panel, combining the May 2015 release of the 1000 Genomes Phase 3 haplotypes and the UK10K imputation reference panel. Data for each genotyping platform were phased and imputed separately. Variants that were only genotyped on the 'V1' platform were flagged due to small sample size, and variants on chrM or chrY, because many of these are not currently called reliably. Using trio data, variants that failed a test for parent–offspring transmission were also flagged; specifically, the child's allele count was regressed against the mean parental allele count and variants with fitted $\beta < 0.6$ and $p < 10^{-20}$ for a test of $\beta<1$ were flagged. Variants with a Hardy–Weinberg $p < 10^{-20}$ in Europeans, or a call rate of < 90%, were also flagged. Genotyped variants were also tested for batch effects and variants with $p < 10^{-50}$ by analysis of variance of genotypes against a factor dividing genotyping date into 20 roughly equal-sized buckets were flagged. For imputed GWAS results, variants with average $r^2 < 0.5$ or minimum $r^2 < 0.3$ in any imputation batch were flagged, as well as SNPs that had strong evidence of an imputation batch effect, using an analysis of variance of the imputed dosages against a factor representing imputation batch; results with $p < 10^{-50}$ were flagged. Each variant flagged by QC on genotyped or imputation data were excluded from the GWAS analysis.

Chinese Reading Study sample

Participants

3,127 Grade 3 to Grade 6 primary students aged nine to 14 years were recruited from three cities and four districts in China (Xi'an-YT, Xi'an-CB, Qingyang, and Baotou). In total, 2,476 participants were eligible for subsequent genotyping and association analysis. Ethical approval was obtained for each cohort at the local level and written informed consent was obtained from all the participants' parents.

Phenotypic measures

Reading accuracy: A Chinese character recognition test was employed to measure each child's reading accuracy [3-5]. The test consisted of 150 single Chinese characters selected from China's Elementary School Textbooks (1996). The average frequency of the characters was 182 per million (ranging from 0 to 2,282), and the reliability of this test was 0.95 [3]. Each child was individually tested and was required to read aloud each character at a time.

Reading fluency: A word list reading task [3] was used to measure each child's reading fluency. In this task, children were asked to name a list of 180 two-character words as rapidly and accurately as possible. All these words were from primary school textbooks and have been learned before Grade 3, such as "我们 (we)" and "太阳 (sun)". The mean frequency of these words was 212.77 per million [6]. Since words included in this task were all simple, this task was administrated to test children's reading fluency. The total time for naming the whole word list was recorded as the measurement of reading fluency.

Genotype quality control, imputation, and analysis

DNA was extracted from saliva samples, and individuals were genotyped using the Illumina Asian screening array (650K) by Beijing Compass Biotechnology. Quality control was performed using standard quality control metrics. Eight samples were excluded as they had sex discrepancies between the records and the genetically inferred data [7,8]. Next, we removed 53 samples who had unexpected duplicates or probable relatives (PI-HAT > 0.20). Then, SNPs were filtered out if they showed a variant call rate < 0.95, a minor allele frequency (MAF) < 0.01, a missing genotype data (mind) < 0.90, or a Hardy-Weinberg Equilibrium (HWE) $p < 10^{-5}$ within each dataset.

For imputation, autosomal variants were aligned to the 1000G genomes phase 1v3 reference panel. Imputation was performed using the Michigan imputation Server 4.0 in 5Mb chunks with 500kb buffers, filtering out variants that were monomorphic in the Genome Asia Pilot (GAsP). Chunks with 51% genotyped variants or concordance rate < 0.92 were fused with neighbouring chunks and re-imputed. Finally, imputed variants were filtered out for $r^2 < 0.60$, MAF < 0.02, mind < 0.1, HWE $p < 10^{-}$

[5] using Plink (v1.90). After quality control procedures had been performed, 2,415 children with 4,261,603 SNPs were included in the final analysis. Association analyses were performed using PLINK, fitting an additive model to the linear regression model with adjustment for sex, age, and the first two principal components [8].

Biological annotations

Genome-wide significant variants and the closest gene(s) were annotated using external reference data through FUMA v1.3.6a [9] (unless otherwise specified) and evaluated for functional or regulatory impact. Specifically, we considered the following annotations of SNPs reaching genome-wide significance ($p < 5 \times 10^{-8}$) (Supplementary Table 10):

- **Gene context:**
    - **Distance:** The distance of the variant to the nearest gene in kb. Variants within the gene body or 1 kb up- or downstream of the transcription start site (TSS) or transcription end site (TES) have a value of zero.
    - **Function:** Whether a variant is intergenic or the functional region in which the variant is located within a gene or RNA locus (e.g., 5' UTR).
- **Combined Annotation Dependent Depletion (CADD) score:** A score of the deleteriousness of variants computed from 63 integrated annotations [10]. The higher the score, the more deleterious a variant is: 12.37 is the threshold indicated by the study of potentially actionable exonic pathogenic single-nucleotide variants in European- and African ancestry patients [11].
- **RegulomeDB category (RDB):** A variant classification system in which variants are grouped according to evidence of having a functional consequence from Category 6 (minimal evidence) to Category 1a (likely to affect binding and linked to expression of a gene target) [12].
- **Chromatin state:** The minimum and the most common 15-core chromatin state across 127 tissue/cell types predicted by ChromHMM [13] from 15 (quiescent/low) to 1 (active TSS).

- **GWAS Catalog:** SNP-trait associations reported in the NHGRI-EBI Catalog of human GWAS [14], including for each variant: the trait(s), the effect allele(s), the PubMed ID(s), the study title(s) and the study sample size(s) (Supplementary Table 2).

And the following annotations of genes which were significant in genome-wide gene-based tests (Supplementary Table 12):

- **Probability of Loss-of-function Intolerance (pLI) score:** A score of intolerance to functional mutation from the ExAC database [15] ranging from zero to one. The closer the score is to one, the more intolerant the gene is to loss-of-function mutations. The threshold suggested by Lek, et al. [15] for likely disease-causing variants is ≥ 0.9.
- **Non-coding Residual Variation Intolerance Score (ncRVIS):** A score of intolerance to mutation to non-coding variants [16]. Where ncRVIS is zero, the gene has the average number of non-coding variants given its total mutational burden; when ncRVIS is greater than zero, the gene has less non-coding variation than expected; when ncRVIS is less than zero, it has more. The ncRVIS percentile reflects the rank of the gene amongst all genes. The more negative the ncRVIS, or the lower the percentile, the more intolerant to non-coding variation the gene is.
- **Residual Variation Intolerance Score (ncRVIS) percentile:** As for ncRVIS score but the percentile of the average RVIS score for the whole gene sequence.
- **Non-coding Genomic Evolutionary Rate Profiling (ncGERP) score:** Identifies constraint in non-coding regions by quantifying deficits in substitutions [16]. It is calculated by taking the average GERP++ score (see Davydov, et al. [17]) across the non-coding sequence. The higher the ncGERP score, the fewer substitutions are present than what would be expected as a result of a neutral rate of evolution, and thus the more conserved are the non-coding regions of the gene. The ncGERP percentile reflects the rank of the gene amongst all genes.

- **Protein-coding Genomic Evolutionary Rate Profiling (pcGERP) percentile:** As for ncGERP score but the percentile of the average GERP score for protein-coding sequence [16].
- **Non-coding Combined Annotation Dependent Depletion (CADD) score:** As for CADD score but the average variant score across the non-coding sequence of the gene [16].
- **Non-coding Genome-Wide Annotation of Variants (ncGWAVA) score:** Predicts the combined functionality of non-coding variants across non-coding sequence [16]. It is the average GWAVA score (see Ritchie, et al. [18]) of variants in the non-coding sequence, ranging from zero to one. The closer ncGWAVA is to one, the more likely the variants in non-coding regions of the gene are functional.
- **Expression in the brain:** Average log2 expression in transcripts per million (TPM) per tissue type per gene from the GTEx v8 dataset [19] for 12 brain tissues: Amygdala, Anterior Cingulate Cortex, Caudate Basal Ganglia, Cerebellar Hemisphere, Cerebellum, Cortex, Frontal Cortex, Hippocampus, Hypothalamus, Nucleus Accumbens Basal Ganglia, Putamen Basal Ganglia, and Substantia Nigra (Supplementary Table 15).

Partitioned heritability

Evolutionary analysis

Enrichment of heritability was estimated for the following evolutionary annotations (as described in Tilot, et al. [20]):

- **Human Gained Enhancers and Promoters:** These regulatory regions were identified based on differential H3K27ac and H3K4me2 patterns in the adult and foetal brain tissues of humans, macaques and mice [19, 20], and shown to be present to a significantly lesser degree in macaques and mice. Thus, these regulatory elements were gained in the last 30 million years of human evolution and may be involved in the emergence of human-specific traits [21,22].

- **Ancient selective sweep regions:** These consist of unusually long genomic regions that reached fixation in human populations possibly due to adaptive advantages in the last 250-650 thousand years [23].
- **Neanderthal-introgressed SNPs:** The genomic variants introduced into the human genome by the admixture of *Homo sapiens* and Neanderthal populations around 50-60,000 years ago [24].
- **Neanderthal Depleted Regions:** Large regions in the human genome that are depleted for Neanderthal ancestry, possibly due to the deleterious effect of the archaic sequences in hybrid individuals [25].

REFERENCES

1. Durand, E.Y. *et al.* A scalable pipeline for local ancestry inference using tens of thousands of reference haplotypes. *bioRxiv*, 2021.01.19.427308 (2021).

2. Henn, B.M. *et al.* Cryptic Distant Relatives Are Common in Both Isolated and Cosmopolitan Genetic Samples. *PLOS ONE* **7**, e34267 (2012).

3. Pan, J. & Shu, H. Rapid Automatized Naming and Its Unique Contribution to Reading: Evidence from Chinese Dyslexia. *Reading development and difficulties in monolingual and bilingual Chinese children*, 125-138 (2014).

4. Lei, L. *et al.* Developmental trajectories of reading development and impairment from ages 3 to 8 years in Chinese children. *Journal of Child Psychology and Psychiatry* **52**, 212-220 (2011).

5. Song, S., Zhang, Y., Shu, H., Su, M. & McBride, C. Universal and Specific Predictors of Chinese Children With Dyslexia - Exploring the Cognitive Deficits and Subtypes. *Frontiers in psychology* **10**, 2904-2904 (2020).

6. Wang, H., Chang, B. R., Li, Y. S., Lin, L. H., Liu, J., Sun, Y. L., et al. . *Modern Chinese Frequency Dictionary*, (Beijing Language Colleague Press, Beijing, 1986).

7. Anderson, C.A. *et al.* Data quality control in genetic case-control association studies. *Nat Protoc* **5**, 1564-73 (2010).

8. Chang, C.C. *et al.* Second-generation PLINK: rising to the challenge of larger and richer datasets. *Gigascience* **4**, 7 (2015).

9. Watanabe, K., Taskesen, E., van Bochoven, A. & Posthuma, D. Functional mapping and annotation of genetic associations with FUMA. *Nat Commun* **8**, 1826 (2017).

10. Kircher, M. *et al.* A general framework for estimating the relative pathogenicity of human genetic variants. *Nature genetics* **46**, 310-315 (2014).

11. Amendola, L.M. *et al.* Actionable exomic incidental findings in 6503 participants: challenges of variant classification. *Genome research* **25**, 305-315 (2015).

12. Boyle, A.P. *et al.* Annotation of functional variation in personal genomes using RegulomeDB. *Genome Res* **22**, 1790-7 (2012).

13. Ernst, J. & Kellis, M. ChromHMM: automating chromatin-state discovery and characterization. *Nature Methods* **9**, 215-216 (2012).

14. Buniello, A. *et al.* The NHGRI-EBI GWAS Catalog of published genome-wide association studies, targeted arrays and summary statistics 2019. *Nucleic Acids Res* **47**, D1005-d1012 (2019).

15. Lek, M. *et al.* Analysis of protein-coding genetic variation in 60,706 humans. *Nature* **536**, 285-291 (2016).

16. Petrovski, S. *et al.* The Intolerance of Regulatory Sequence to Genetic Variation Predicts Gene Dosage Sensitivity. *PLOS Genetics* **11**, e1005492 (2015).

17. Davydov, E.V. *et al.* Identifying a High Fraction of the Human Genome to be under Selective Constraint Using GERP++. *PLOS Computational Biology* **6**, e1001025 (2010).

18. Ritchie, G.R.S., Dunham, I., Zeggini, E. & Flicek, P. Functional annotation of noncoding sequence variants. *Nature methods* **11**, 294-296 (2014).

19. Aguet, F. *et al.* The GTEx Consortium atlas of genetic regulatory effects across human tissues. *bioRxiv*, 787903 (2019).

20. Tilot, A.K. *et al.* The Evolutionary History of Common Genetic Variants Influencing Human Cortical Surface Area. *Cerebral Cortex* **31**, 1873-1887 (2020).

21. Vermunt, M.W. *et al.* Epigenomic annotation of gene regulatory alterations during evolution of the primate brain. *Nat Neurosci* **19**, 494-503 (2016).

22. Reilly, S.K. *et al.* Evolutionary genomics. Evolutionary changes in promoter and enhancer activity during human corticogenesis. *Science* **347**, 1155-9 (2015).

23. Peyrégne, S., Boyle, M.J., Dannemann, M. & Prüfer, K. Detecting ancient positive selection in humans using extended lineage sorting. *Genome Res* **27**, 1563-1572 (2017).

24. Vernot, B. & Akey, J.M. Resurrecting surviving Neandertal lineages from modern human genomes. *Science* **343**, 1017-21 (2014).

25. Vernot, B. *et al.* Excavating Neandertal and Denisovan DNA from the genomes of Melanesian individuals. *Science* **352**, 235-239 (2016).

26. Bates, T.C. *et al.* Dyslexia and DYX1C1: deficits in reading and spelling associated with a missense mutation. *Molecular Psychiatry* **15**, 1190-6 (2010).

27. Brkanac, Z. *et al.* Evaluation of candidate genes for DYX1 and DYX2 in families with dyslexia. *American Journal of Medical Genetics Part B: Neuropsychiatric Genetics* **144b**, 556-60 (2007).

28. Carrion-Castillo, A. *et al.* Association analysis of dyslexia candidate genes in a Dutch longitudinal sample. *European Journal of Human Genetics* **25**, 452-460 (2017).

29. Chen, Y., Zhao, H., Zhang, Y.X. & Zuo, P.X. DCDC2 gene polymorphisms are associated with developmental dyslexia in Chinese Uyghur children. *Neural Regeneration Research* **12**, 259-266 (2017).

30. Cope, N.A. *et al.* No support for association between Dyslexia Susceptibility 1 Candidate 1 and developmental dyslexia. *Molecular Psychiatry* **10**, 237-238 (2005).

31. Couto, J.M. *et al.* The KIAA0319-like (KIAA0319L) gene on chromosome 1p34 as a candidate for reading disabilities. *Journal of Neurogenetics* **22**, 295-313 (2008).

32. Couto, J.M. *et al.* Association of reading disabilities with regions marked by acetylated H3 histones in KIAA0319. *American Journal of Medical Genetics Part B: Neuropsychiatric Genetics* **153b**, 447-462 (2010).

33. Dahdouh, F. *et al.* Further evidence for DYX1C1 as a susceptibility factor for dyslexia. *Psychiatric Genetics* **19**, 59-63 (2009).

34. Francks, C. *et al.* A 77-kilobase region of chromosome 6p22.2 is associated with dyslexia in families from the United Kingdom and from the United States. *American Journal of Human Genetics* **75**, 1046-58 (2004).

35. Harold, D. *et al.* Further evidence that the KIAA0319 gene confers susceptibility to developmental dyslexia. *Molecular Psychiatry* **11**, 1085-1091 (2006).

36. Kong, R. *et al.* Genetic variant in DIP2A gene is associated with developmental dyslexia in Chinese population. *American Journal of Medical Genetics Part B: Neuropsychiatric Genetics* **171**, 203-208 (2016).

37. Lind, P.A. *et al.* Dyslexia and DCDC2: normal variation in reading and spelling is associated with DCDC2 polymorphisms in an Australian population sample. *European Journal of Human Genetics* **18**, 668-73 (2010).

38. Mascheretti, S. *et al.* KIAA0319 and ROBO1: evidence on association with reading and pleiotropic effects on language and mathematics abilities in developmental dyslexia. *Journal of Human Genetics* **59**, 189-197 (2014).

39. Matsson, H. *et al.* Polymorphisms in DCDC2 and S100B associate with developmental dyslexia. *Journal of Human Genetics* **60**, 399-401 (2015).

40. Mozzi, A. *et al.* A common genetic variant in FOXP2 is associated with language-based learning (dis)abilities: Evidence from two Italian independent samples. *American Journal of Medical Genetics Part B: Neuropsychiatric Genetics* **174B**, 578– 586 (2017).

41. Müller, B. *et al.* ATP2C2 and DYX1C1 are putative modulators of dyslexia-related MMR. *Brain and Behavior* **7**, e00851-e00851 (2017).

42. Müller, B. *et al.* Association, characterisation and meta-analysis of SNPs linked to general reading ability in a German dyslexia case-control cohort. *Scientific Reports* **6**, 27901 (2016).

43. Newbury, D.F. *et al.* Investigation of dyslexia and SLI risk variants in reading- and language-impaired subjects. *Behavior Genetics* **41**, 90-104 (2011).

44. Newbury, D.F. *et al.* CMIP and ATP2C2 modulate phonological short-term memory in language impairment. *American Journal of Human Genetics* **85**, 264-272 (2009).

45. Paracchini, S. *et al.* Analysis of dyslexia candidate genes in the Raine cohort representing the general Australian population. *Genes, Brain and Behavior* **10**, 158-65 (2011).

46. Peter, B. *et al.* Replication of CNTNAP2 association with nonword repetition and support for FOXP2 association with timed reading and motor activities in a dyslexia family sample. *Journal of Neurodevelopmental Disorders* **3**, 39-49 (2011).

47. Poelmans, G. *et al.* Identification of novel dyslexia candidate genes through the analysis of a chromosomal deletion. *American Journal of Medical Genetics Part B: Neuropsychiatric Genetics* **150B**, 140-7 (2009).

48. Scerri, T.S. *et al.* Putative functional alleles of DYX1C1 are not associated with dyslexia susceptibility in a large sample of sibling pairs from the UK. *Journal of Medical Genetics* **41**, 853-7 (2004).

49. Scerri, T.S. *et al.* DCDC2, KIAA0319 and CMIP are associated with reading-related traits. *Biological Psychiatry* **70**, 237-45 (2011).

50. Schumacher, J. *et al.* Strong genetic evidence of DCDC2 as a susceptibility gene for dyslexia. *American Journal of Human Genetics* **78**, 52-62 (2006).

51. Shao, S. *et al.* The Roles of Genes in the Neuronal Migration and Neurite Outgrowth Network in Developmental Dyslexia: Single- and Multiple-Risk Genetic Variants. *Molecular Neurobiology* **53**, 3967-3975 (2016).

52. Sun, X. *et al.* ROBO1 polymorphisms, callosal connectivity, and reading skills. *Human Brain Mapping* **38**, 2616-2626 (2017).

53. Taipale, M. *et al.* A candidate gene for developmental dyslexia encodes a nuclear tetratricopeptide repeat domain protein dynamically regulated in brain. *Proceedings of the National Academy of Sciences of the United States of America* **100**, 11553-8 (2003).

54. Tolosa, A. *et al.* FOXP2 gene and language impairment in schizophrenia: association and epigenetic studies. *BMC Medical Genetics* **11**, 114-114 (2010).

55. Tran, C. *et al.* Association of the ROBO1 gene with reading disabilities in a family-based analysis. *Genes, Brain and Behavior* **13**, 430-438 (2014).

56. Venkatesh, S.K., Siddaiah, A., Padakannaya, P. & Ramachandra, N.B. Lack of association between genetic polymorphisms in ROBO1, MRPL19/C2ORF3 and THEM2 with developmental dyslexia. *Gene* **529**, 215-9 (2013).

57. Vernes, S.C. *et al.* A functional genetic link between distinct developmental language disorders. *New England Journal of Medicine* **359**, 2337-2345 (2008).

58. Whitehouse, A.J.O., Bishop, D.V.M., Ang, Q.W., Pennell, C.E. & Fisher, S.E. CNTNAP2 variants affect early language development in the general population. *Genes, Brain and Behavior* **10**, 451-456 (2011).

59. Wigg, K.G. *et al.* Support for EKN1 as the susceptibility locus for dyslexia on 15q21. *Molecular Psychiatry* **9**, 1111-21 (2004).

60. Gialluisi, A. *et al.* Genome-wide association scan identifies new variants associated with a cognitive predictor of dyslexia. *Translational Psychiatry* **9**, 77 (2019).

GENLANG QUANTITATIVE TRAIT CONSORTIUM ACKNOWLEDGEMENTS

General Acknowledgements

BM, BMM, BSP, CF, EE, EV, GA, MvD and SEF are supported by the Max Planck Society. AG and TFMA were supported by the Munich Cluster for Systems Neurology (SyNergy), and AG was supported by Fondazione Umberto Veronesi. ATM is supported by the National Health and Medical Research Council of Australia (NHMRC) Fellowships (1105008; 1195955) and Centre of Research Excellence grant (1116976). AJOW is supported by an Investigator Grant from the NHMRC (1173896). BSP and MvD are supported by the Simons Foundation Autism Research Initiative grant (514787 to BSP). CYS works in the Medical Research Council Integrative Epidemiology Unit at the University of Bristol (MC_UU_00011/3). DIB acknowledges the Royal Netherlands Academy of Science Professor Award (PAH/6635). EE is supported by a National Institutes of Health (NIH) R01 grant (R01DC016977). EGW is supported by NICHD P50 HD 27802. FR is supported by Agence Nationale de la Recherche (ANR-06-NEURO-019-01, ANR-17-EURE-0017 IEC, ANR-10-IDEX-0001-02 PSL, ANR-11-BSV4-014-01), European Commission (LSHM-CT-2005-018696). HT is supported by a VICI grant of the Netherlands Organization for Scientific Research (NWO) and The Netherlands Organisation for Health Research and Development (ZonMW) (016.VICI.170.200). JCDF was supported by the National Institute of Child Health and Human Development (NICHD) (P50 HD 27802). JJM, JBTo, and TK were supported by the National Institutes of Health (NIH) (R01 DC014489). KP was supported by the Hospital for Sick Children Research Training Program (Restracomp). KR is supported by a Sir Henry Wellcome Postdoctoral Fellowship. MJS is supported by a Wellcome Trust Programme grant (WT082032MA). SP and FA are supported by the Royal Society (UF150663; RGF\EA\180141). TB is supported by Institut Pasteur, the Bettencourt-Schueller Foundation, Université de Paris.

We are extremely grateful to all the children, twins, families and participants who took part and are taking part in the 22 cohorts whose data contributed to these GWAS meta-analyses. We also thank the staff working on the different cohorts, including volunteers, study coordinators, interviewers, teachers, nurses, research scientists, general practitioners, midwives, psychologists, psychometrists,

computer and laboratory technicians and colleagues who assisted in the quality control and preparation of the imputed GWAS data and the pharmacies and hospitals that were involved.

Adolescent Brain Cognitive Development ^{SM} Study

Data used in the preparation of this article were obtained from the Adolescent Brain Cognitive Development^{SM} (ABCD) Study (https://abcdstudy.org), held in the NIMH Data Archive (NDA). This is a multisite, longitudinal study designed to recruit more than 10,000 children age 9-10 and follow them over 10 years into early adulthood. The ABCD Study® is supported by the NIH and additional federal partners under award numbers U01DA041048, U01DA050989, U01DA051016, U01DA041022, U01DA051018, U01DA051037, U01DA050987, U01DA041174, U01DA041106, U01DA041117, U01DA041028, U01DA041134, U01DA050988, U01DA051039, U01DA041156, U01DA041025, U01DA041120, U01DA051038, U01DA041148, U01DA041093, U01DA041089, U24DA041123, U24DA041147. A full list of supporters is available at https://abcdstudy.org/federal-partners.html. A listing of participating sites and a complete listing of the study investigators can be found at https://abcdstudy.org/consortium_members/. ABCD consortium investigators designed and implemented the study and/or provided data but did not necessarily participate in the analysis or writing of this report. This manuscript reflects the views of the authors and may not reflect the opinions or views of the NIH or ABCD consortium investigators. The ABCD data used in this report are summarized in this NDA study (https://doi.org/10.15154/1522876).

Aston cohort

Data collection for the Aston Cohort has been supported by funding from the European Union Horizon 2020 Programme (641652), The Waterloo Foundation (797/17290), and with the invaluable support of the participants and their families. Data analyses were supported by the St Andrews Bioinformatics Unit which is funded by Wellcome Trust ISSF awards (105621/Z/14/Z and 204821/Z/16/Z).

Avon Longitudinal Study of Parents and their Children (ALSPAC)

We are extremely grateful to all the families who took part in this study, the midwives for their help in recruiting them, and the whole ALSPAC team, which includes interviewers, computer and laboratory

technicians, clerical workers, research scientists, volunteers, managers, receptionists and nurses. GWAS data were generated by Sample Logistics and Genotyping Facilities at Wellcome Sanger Institute and LabCorp (Laboratory Corporation of America) using support from 23andMe. The UK Medical Research Council and Wellcome (Grant ref: 217065/Z/19/Z) and the University of Bristol provide core support for ALSPAC. A comprehensive list of grants funding is available on the ALSPAC website (http://www.bristol.ac.uk/alspac/external/documents/grant-acknowledgements.pdf). The publication is the work of the authors and they will serve as guarantor for the ALSPAC contribution to this article.

The Basque Center on Cognition, Brain and Language (BCBL)

Data collection was supported by the Basque Government through the BERC program, and by the Agencia Estatal de Investigación through BCBL Severo Ochoa excellence accreditation.

Brisbane Adolescent Twins Study (BATS)

The research was supported by the Australian Research Council (A7960034, A79906588, A79801419, DP0212016 and DP0343921), with genotyping funded by the NHMRC (Medical Bioinformatics Genomics Proteomics Program, 389891).

Colorado Learning Disabilities Research Center (CLDRC)

The CLDRC was supported by a NICHD grant P50 HD 27802.

The Early Language in Victoria Study (ELVS)

Funding by the NHMRC grant number 436958 is gratefully appreciated.

Familial Influences on Literacy Abilities (FIOLA)

The FIOLA cohort (PIs EvB and PFdJ) is supported by the University of Amsterdam, the MPI Nijmegen, and by fellowships awarded to EvB (NWO's Rubicon 446-12-005 and VENI 451-15-017). We are grateful to the NEMO Science Museum.

Generation R

The Generation R Study is conducted by the Erasmus Medical Center in close collaboration with School of Law and Faculty of Social Sciences of the Erasmus University Rotterdam, the Municipal Health

Service Rotterdam area, the Rotterdam Homecare Foundation, and the Stichting Trombosedienst & Artsenlaboratorium Rijnmond (STAR[1]MDC), Rotterdam.

The Genes, Reading and Dyslexia (GRaD) Study

The GRaD Study was funded by the generous support of the Manton Foundation, the NIH (P50-HD027802; K99-HD094902), and the Lambert Family.

Neurodys

We would like to acknowledge our project partners Catherine Billard, Caroline Bogliotti, Vanessa Bongiovanni, Laure Bricout, Camille Chabernaud, Yves Chaix, Isabelle Comte-Gervais, Florence Delteil-Pinton, Jean-François Démonet, Fabien Fauchereau, Florence George, Christophe-Loïc Gérard, Ferenc Honbolygó, Guillaume Huguet, Stéphanie Iannuzzi, Marie Lageat, Marie-France Leheuzey, Marie-Thérèse Lenormand, Marion Liébert, Emilie Longeras, Emilie Racaud, Isabelle Soares-Boucaud, Sylviane Valdois, Nadège Villiermet, and Johannes Ziegler. This project was funded by the EU FP6 grant to the Neurodys consortium, the SNSF (32-108130) and Austrian Science Fund (18351-B02).

Netherlands Twin Register (NTR)

Funding from the NWO and ZonMW is gratefully acknowledged: Twin family database for behavior genomics studies (NWO 480-04-004); Longitudinal data collection from teachers of Dutch twins and their siblings (481-08-011), Twin-family-study of individual differences in school achievement (NWO-FES, 056-32-010), Genotype-phenotype database for behavior genetic and genetic epidemiological studies (ZonMw Middelgroot 911-09-032); Genetic influences on stability and change in psychopathology from childhood to young adulthood (ZonMW 912-10-020), Why some children thrive (OCW_Gravity program –NWO-024.001.003), Netherlands Twin Registry Repository: researching the interplay between genome and environment (NWO-Groot 480-15-001/674); BBMRI –NL (184.021.007 and 184.033.111): Biobanking and Biomolecular Resources Research Infrastructure; Spinozapremie (NWO- 56-464-14192); Amsterdam Public Health (APH) and Amsterdam Reproduction and Development (AR&D) Research Institutes; European Science Council Genetics of Mental Illness (ERC Advanced, 230374); Aggression in Children: Unraveling gene-environment interplay to inform

Treatment and InterventiON strategies (ACTION) project (European Union Seventh Framework Program (FP7/2007-2013) no 602768); Rutgers University Cell and DNA Repository cooperative agreement (NIMH U24 MH068457-06); Developmental Study of Attention Problems in Young Twins (NIMH, RO1 MH58799-03); Grand Opportunity grant Developmental trajectories of psychopathology (NIMH 1RC2 MH089995) and the Avera Institute for Human Genetics.

Pediatric Imaging, Neurocognition and Genetics Study (PING)

Data collection and sharing for this project was funded by the Pediatric Imaging, Neurocognition and Genetics Study (PING) (NIH grant RC2DA029475). PING is funded by the National Institute on Drug Abuse and the Eunice Kennedy Shriver NICHD. PING data are disseminated by the PING Coordinating Center at the Center for Human Development, University of California, San Diego.

The Philadelphia Neurodevelopmental Cohort (PNC)

Support for the collection of the data sets was provided by grant RC2MH089983 awarded to Raquel Gur and RC2MH089924 awarded to Hakon Hakonarson. All subjects were recruited through the Center for Applied Genomics at The Children's Hospital in Philadelphia. Reading performance was assessed as part of the cognitive test battery and collected by Gur et al. (2012). Genotyping was funded by an Institutional Development Award to the Center for Applied Genomics from The Children's Hospital of Philadelphia and a donation from Adele and Daniel Kubert. We would also like to thank the NIH data repository. The dataset analysed was accessed via dbGaP number 19848 and 12043 (Neurodevelopmental Genomics: Trajectories of Complex Phenotypes (phs000607.v3.p2)).

The Raine Study

The Raine Study was supported by the NHMRC (572613, 403981 and 1059711), the Canadian Institutes of Health Research (MOP-82893), and WA Health, Government of Western Australia (Future Health WA G06302). Funding was also generously provided by Safe Work Australia. The authors gratefully acknowledge the NHMRC for their long-term funding to the study over the last 30 years and also the following institutes for providing funding for Core Management of the Raine Study: The University of Western Australia (UWA), Curtin University, Women and Infants Research Foundation, Telethon Kids

Institute, Edith Cowan University, Murdoch University, The University of Notre Dame Australia and The Raine Medical Research Foundation. This work was supported by resources provided by the Pawsey Supercomputing Centre with funding from the Australian Government and Government of Western Australia.

Saguenay Youth Study (SYS)

The Canadian Institutes of Health Research (ZP, TP), Heart and Stroke Foundation of Quebec (ZP), and the Canadian Foundation for Innovation (ZP) support the Saguenay Youth Study.

SLI Consortium (SLIC)

SLI Consortium members are as follows: Wellcome Trust Centre for Human Genetics, Oxford: D. F. Newbury, N. H. Simpson, F. Ceroni, A. P. Monaco; Max Planck Institute for Psycholinguistics, Nijmegen: S. E. Fisher, C. Francks; Newcomen Centre, Evelina Children's Hospital, St Thomas' Hospital, London: G. Baird, V. Slonims; Child and Adolescent Psychiatry Department and Medical Research Council Centre for Social, Developmental, and Genetic Psychiatry, Institute of Psychiatry, London: P. F. Bolton; Medical Research Council Centre for Social, Developmental, and Genetic Psychiatry Institute of Psychiatry, London: E. Simonoff; Salvesen Mindroom Centre, Child Life & Health, School of Clinical Sciences, University of Edinburgh: A. O'Hare; Cell Biology & Genetics Research Centre, St. George's University of London: J. Nasir; Queen's Medical Research Institute, University of Edinburgh: J. Seckl; Department of Speech and Language Therapy, Royal Hospital for Sick Children, Edinburgh: H. Cowie; Speech and Hearing Sciences, Queen Margaret University: A. Clark, J. Watson; Department of Educational and Professional Studies, University of Strathclyde: W. Cohen; Department of Child Health, the University of Aberdeen: A. Everitt, E. R. Hennessy, D. Shaw, P. J. Helms; Audiology and Deafness, School of Psychological Sciences, University of Manchester: Z. Simkin, G. Conti-Ramsden; Department of Experimental Psychology, University of Oxford: D. V. M. Bishop; Biostatistics Department, Institute of Psychiatry, London: A. Pickles.

The collection and genetic characterisation of SLIC samples was funded by the Wellcome Trust (076566) and the UK Medical Research Council (G1000569).

The Twins Early Development Study (TEDS)

TEDS is supported by a programme grant to RP from the UK Medical Research Council (MR/V012878/1 and previously MR/M021475/1), with additional support from the NIH (AG046938). The research leading to these results has also received funding from the European Research Council under the European Union's Seventh Framework Programme (FP7/2007-2013/ grant agreement number 602768).

Toronto

Support for this project was provided by grants from the Canadian Institutes of Health Research (MOP-133440).

UK Dyslexia

Cohort recruitment and data collection was supported by Wellcome Trust (076566/Z/05/Z and 075491/Z/04) and The Waterloo Foundation (grants to JBTa and SP; 797–1720). Genotype data were generated and analyses funded by the EU (Neurodys, 018696) and the Royal Society (UF100463 grant to SP).

York

The York cohort was funded by Wellcome Trust Programme Grant 082036/B/07/Z.

FIGURES

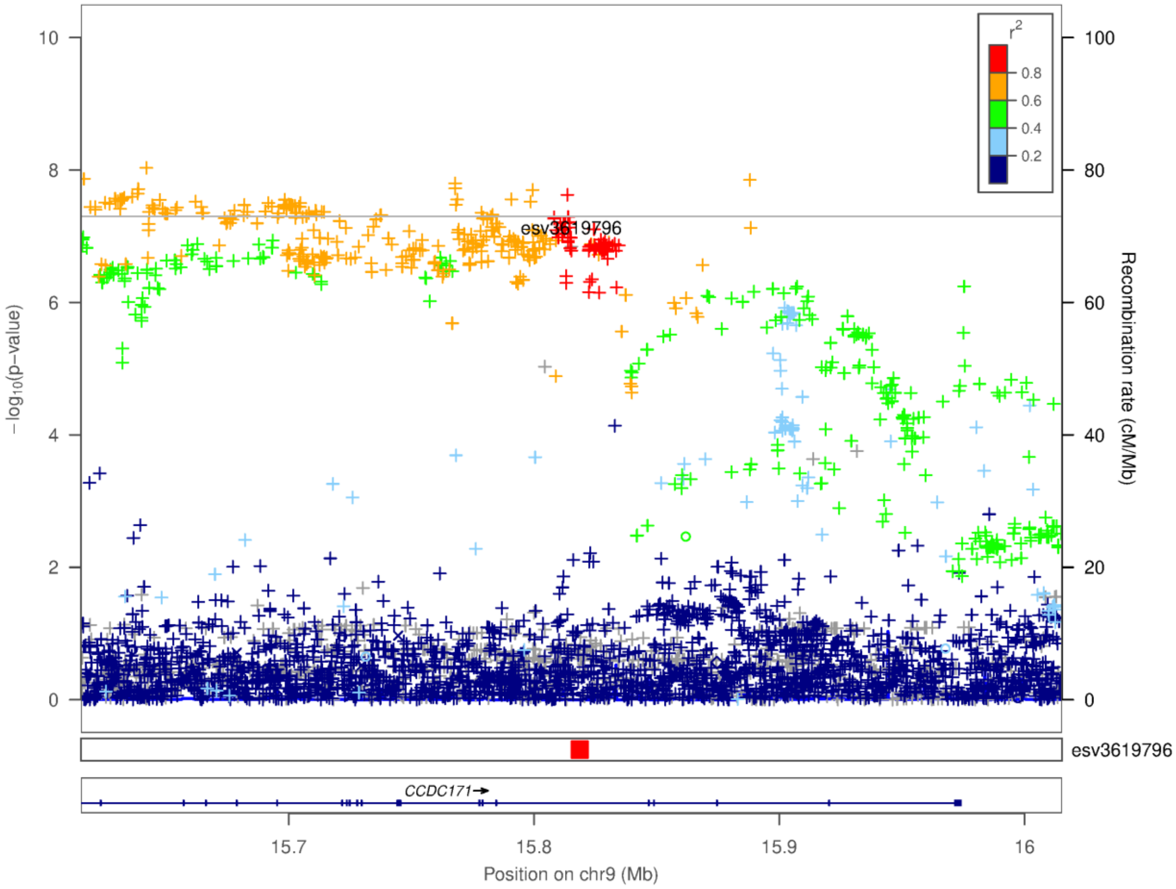

Supplementary Figure 1.i. Regional association plot which includes information on the credible variant set and known genes in the region for the significant association between dyslexia and chr9p22.3 esv3619796 structural variant nearby rs3122702

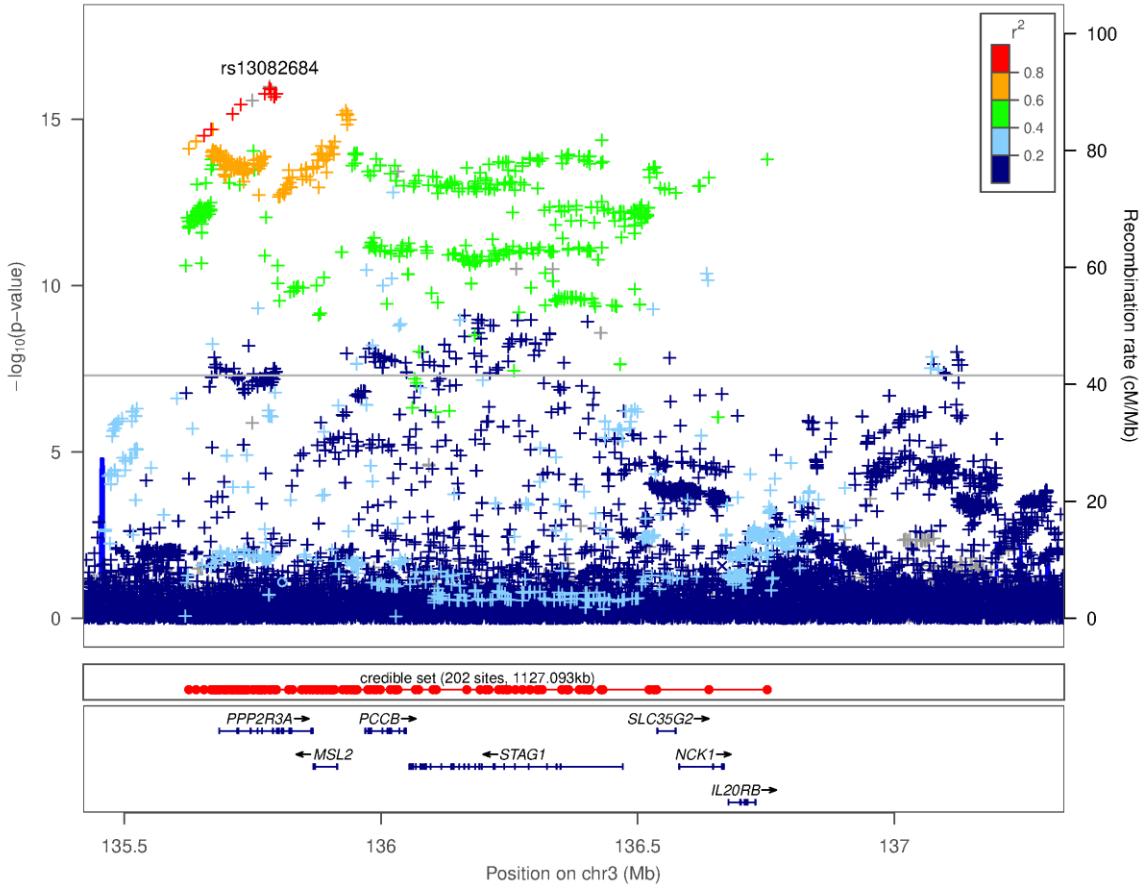

Supplementary Figure 1.ii. Regional association plot which includes information on the credible variant set and known genes in the region for the significant association between dyslexia and chr3q22.3 rs13082684

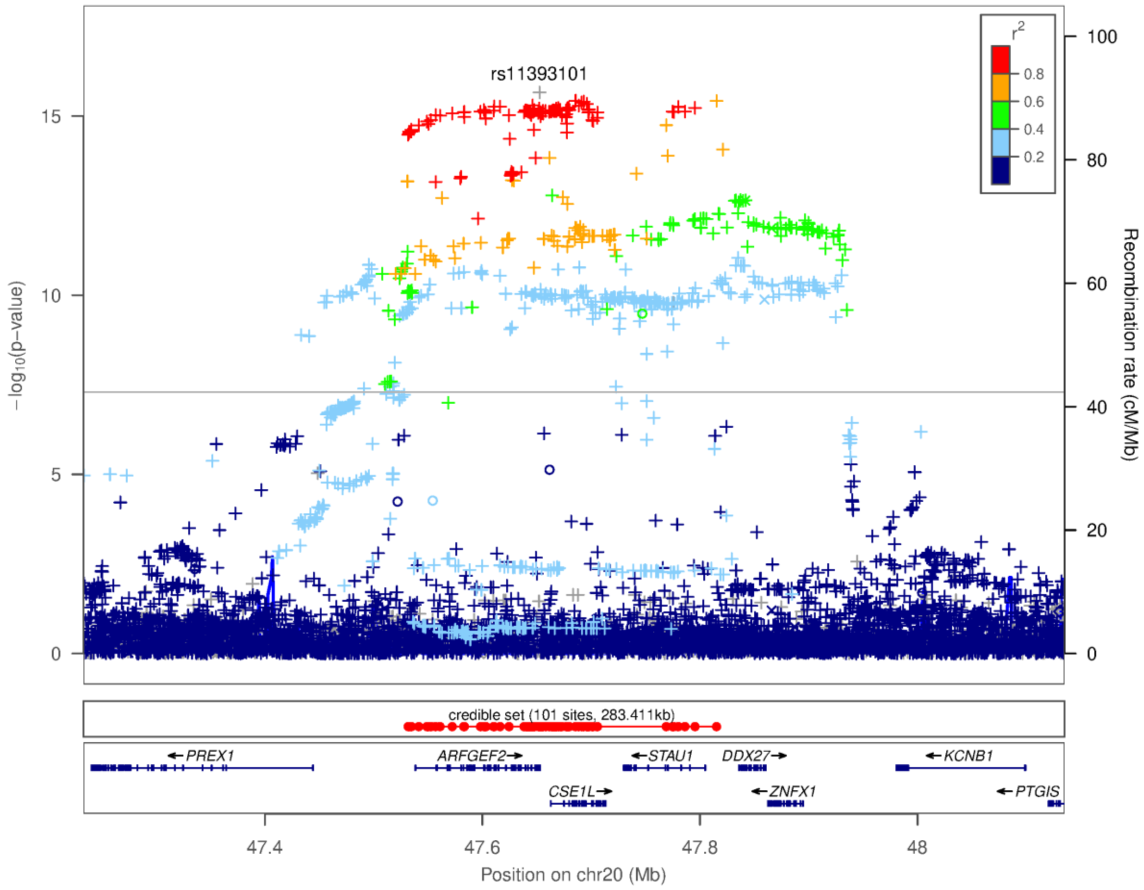

Supplementary Figure 1.iii. Regional association plot which includes information on the credible variant set and known genes in the region for the significant association between dyslexia and chr20q13.13 rs11393101

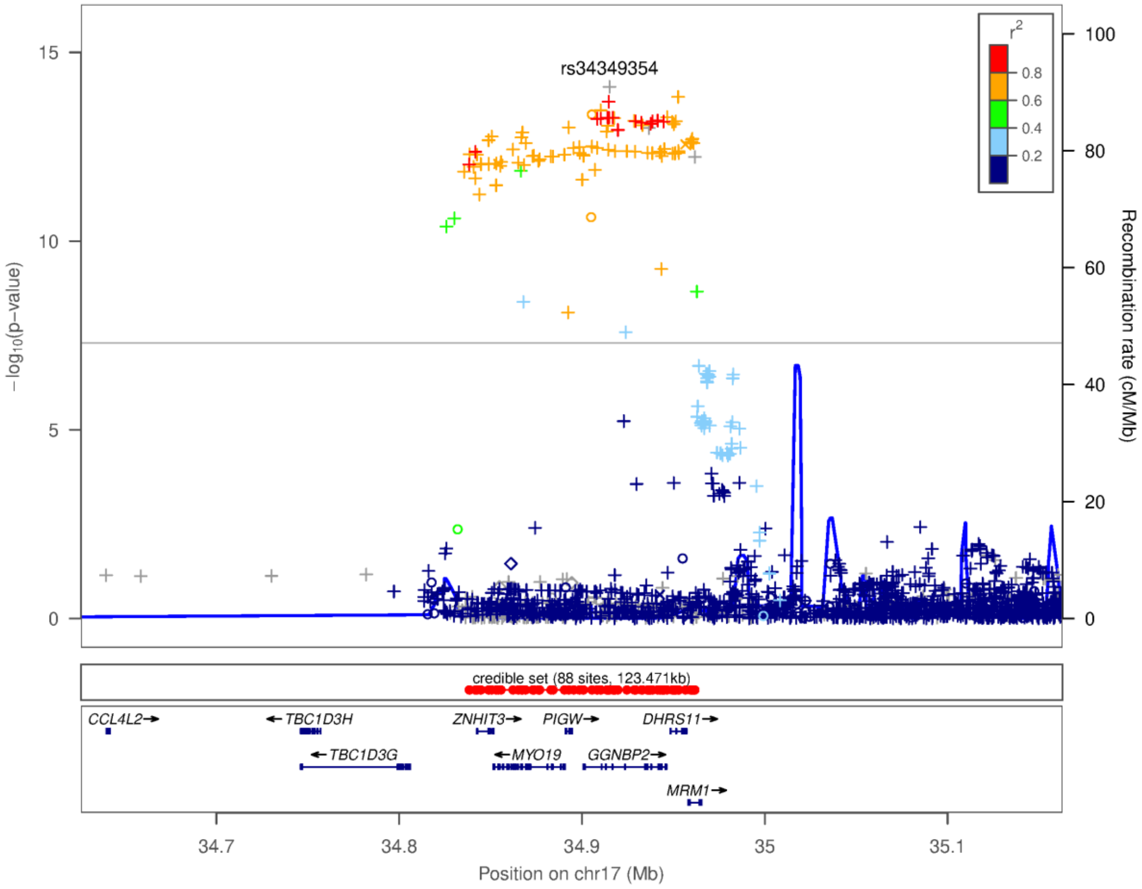

Supplementary Figure 1.iv. Regional association plot which includes information on the credible variant set and known genes in the region for the significant association between dyslexia and chr17q12 rs34349354

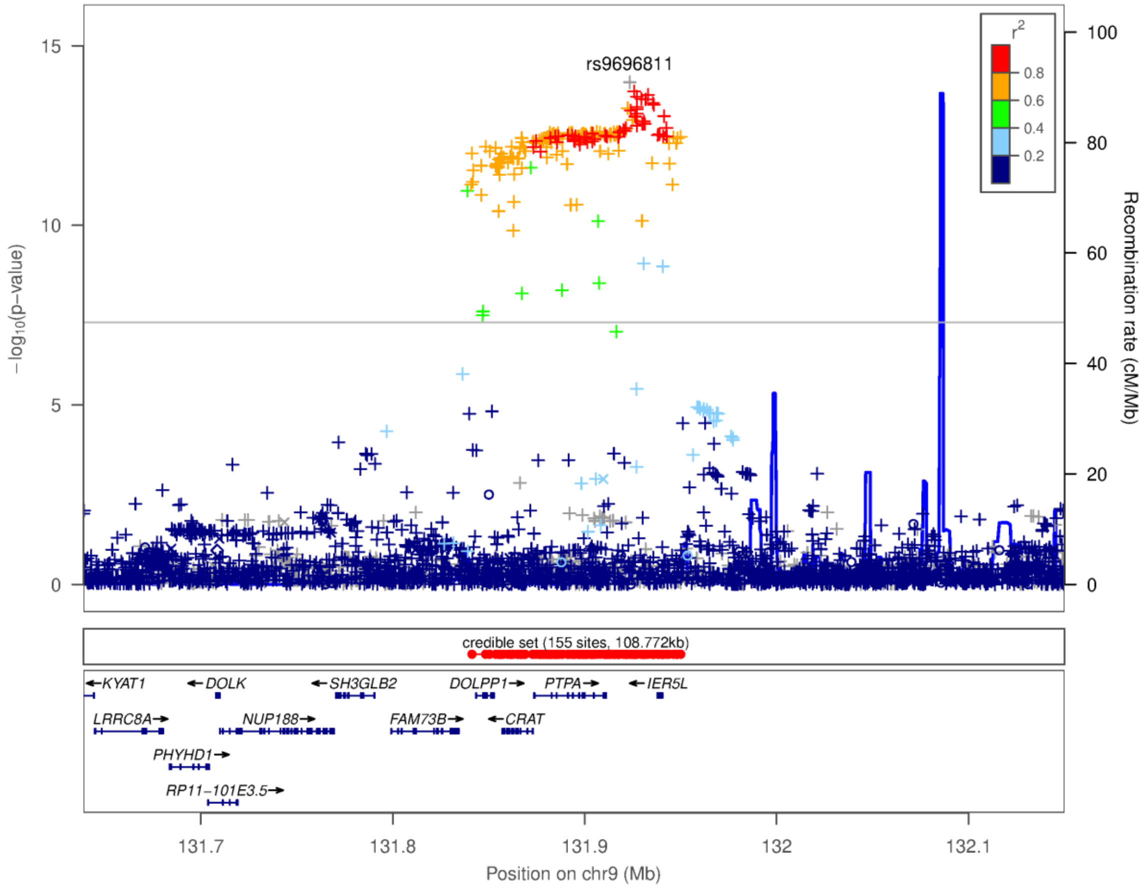

Supplementary Figure 1.v. Regional association plot which includes information on the credible variant set and known genes in the region for the significant association between dyslexia and chr9q34.11 rs9696811

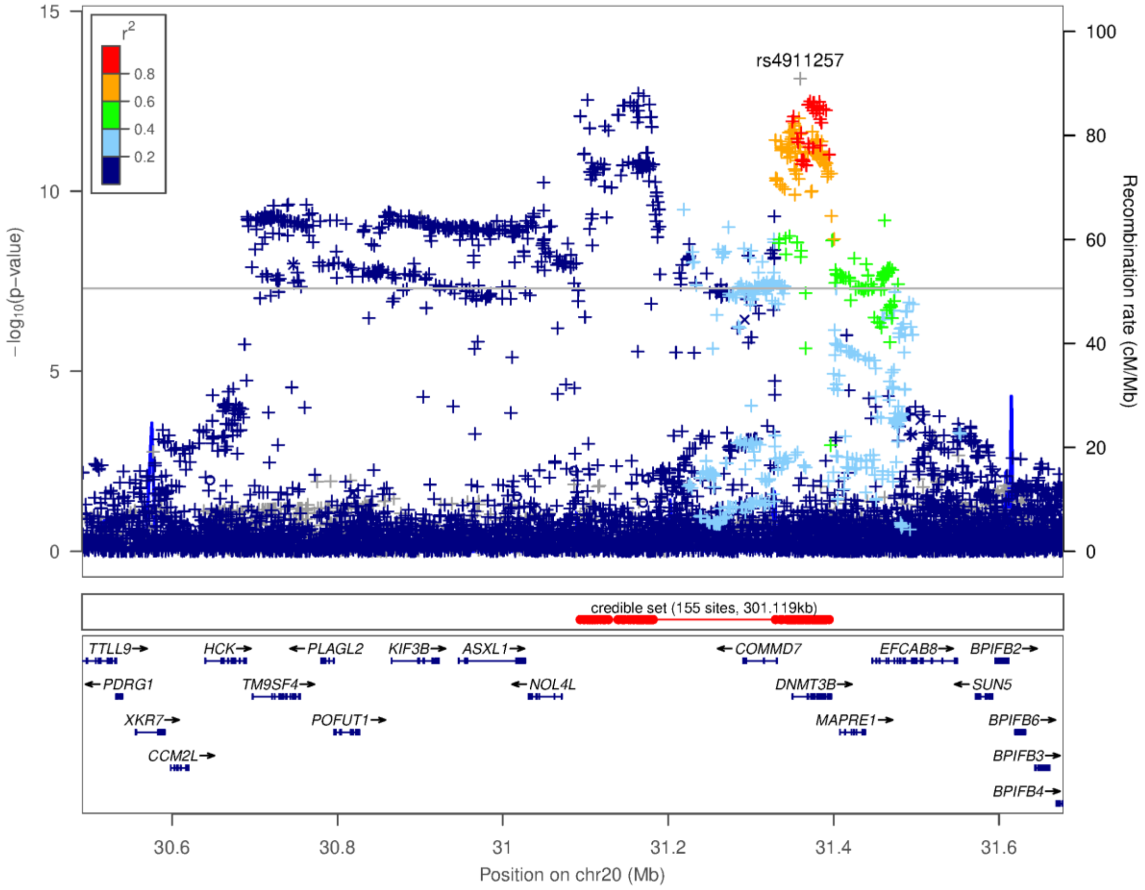

Supplementary Figure 1.vi. Regional association plot which includes information on the credible variant set and known genes in the region for the significant association between dyslexia and chr20q11.21 rs4911257

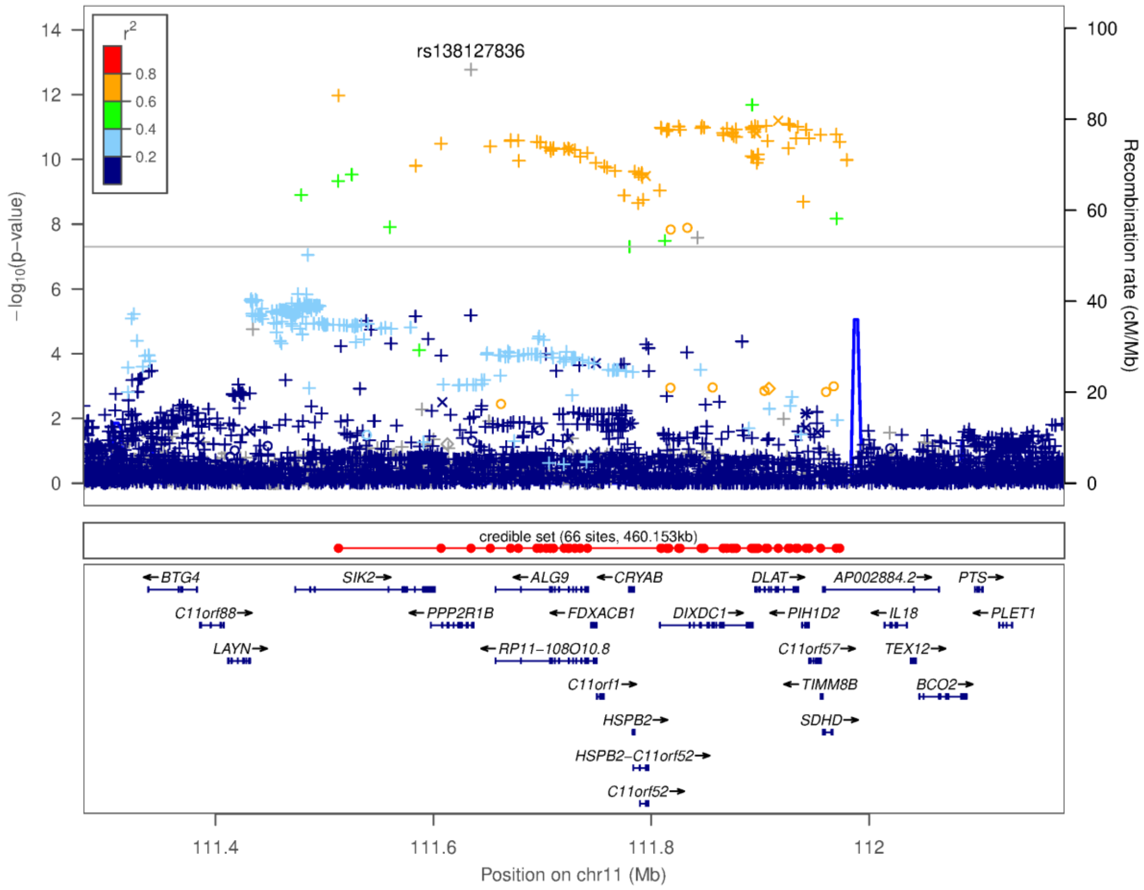

Supplementary Figure 1.vii. Regional association plot which includes information on the credible variant set and known genes in the region for the significant association between dyslexia and chr11q23.1 rs138127836

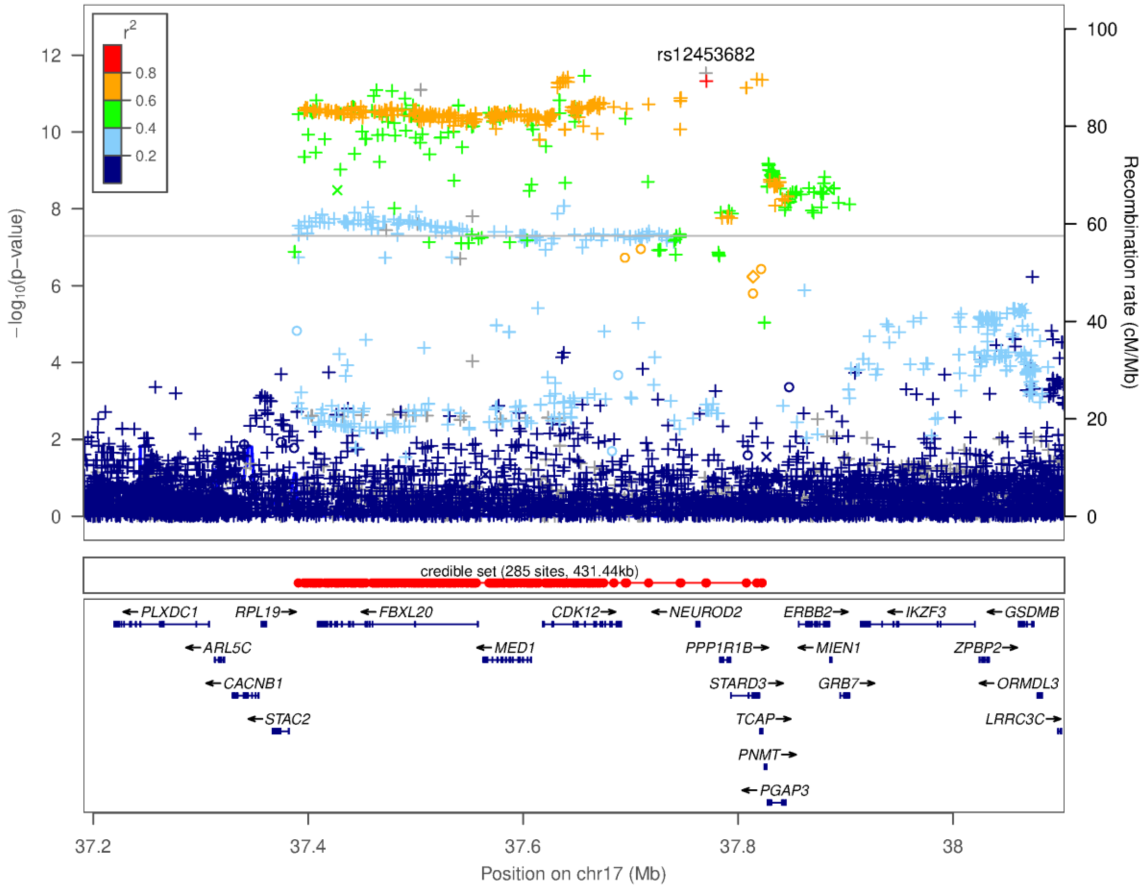

Supplementary Figure 1.viii. Regional association plot which includes information on the credible variant set and known genes in the region for the significant association between dyslexia and chr17q12 rs12453682

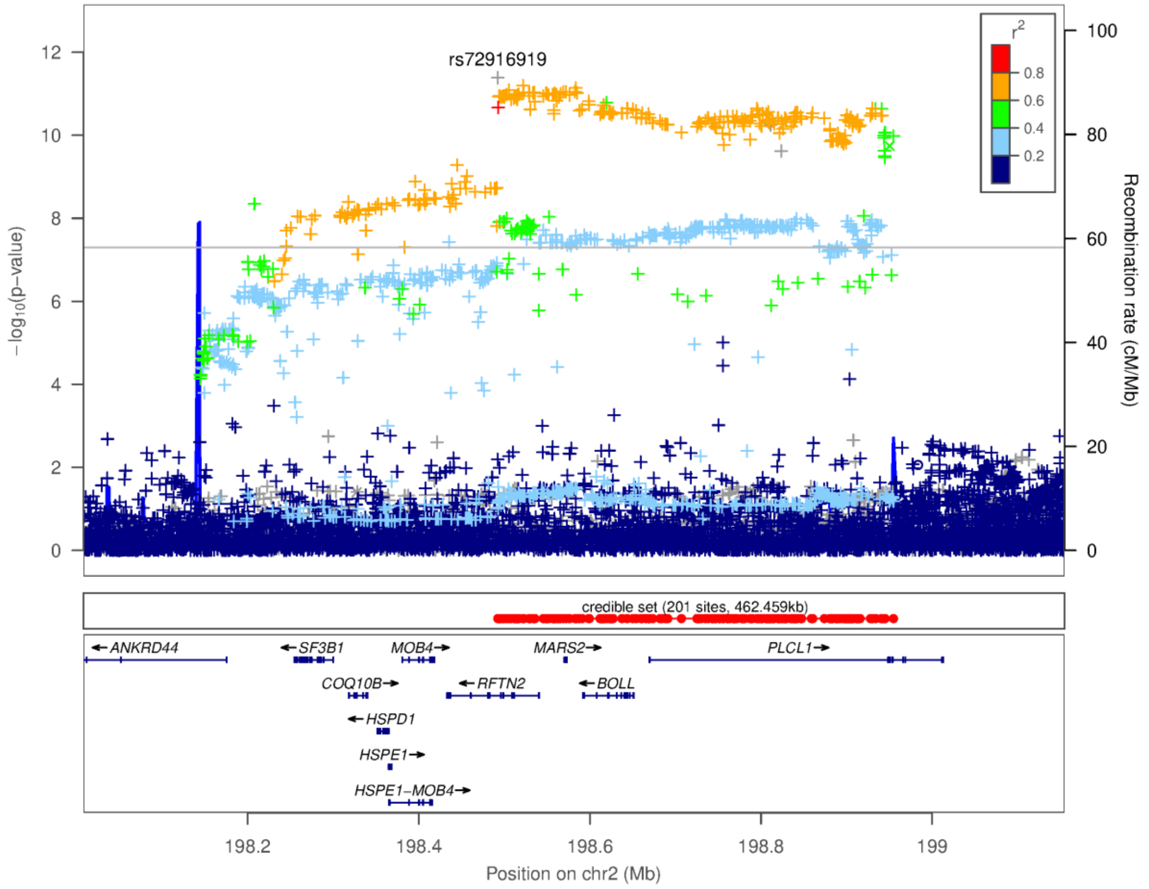

Supplementary Figure 1.ix. Regional association plot which includes information on the credible variant set and known genes in the region for the significant association between dyslexia and chr2q33.1 rs72916919

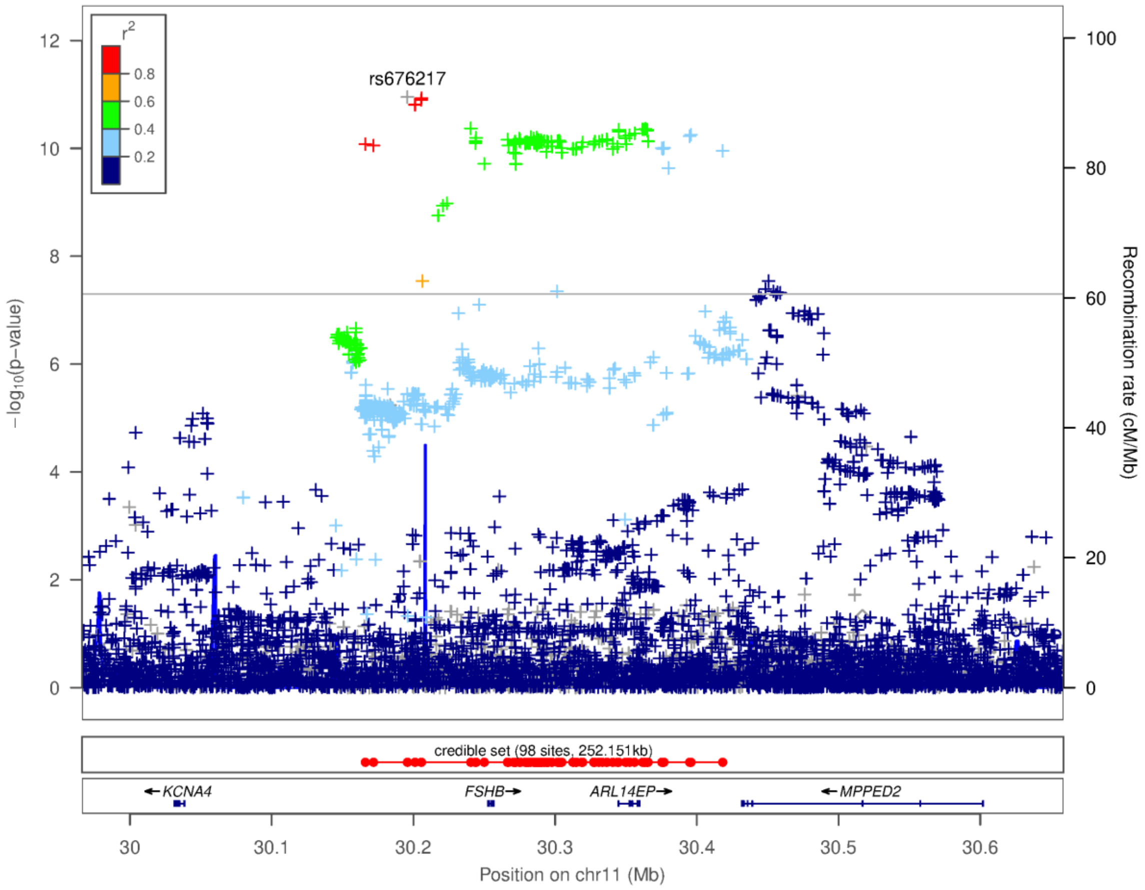

Supplementary Figure 1.x. Regional association plot which includes information on the credible variant set and known genes in the region for the significant association between dyslexia and chr11p14.1 rs676217

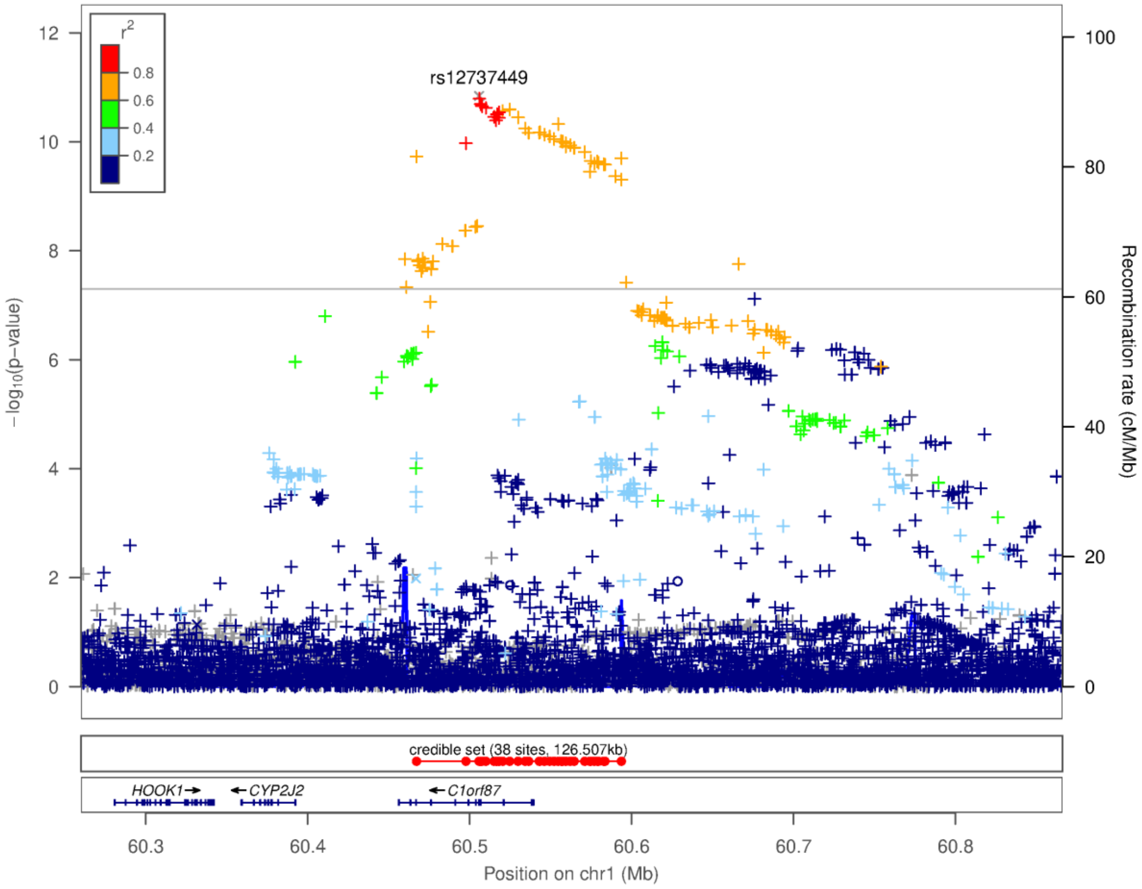

Supplementary Figure 1.xi. Regional association plot which includes information on the credible variant set and known genes in the region for the significant association between dyslexia and chr1p32.1 rs12737449

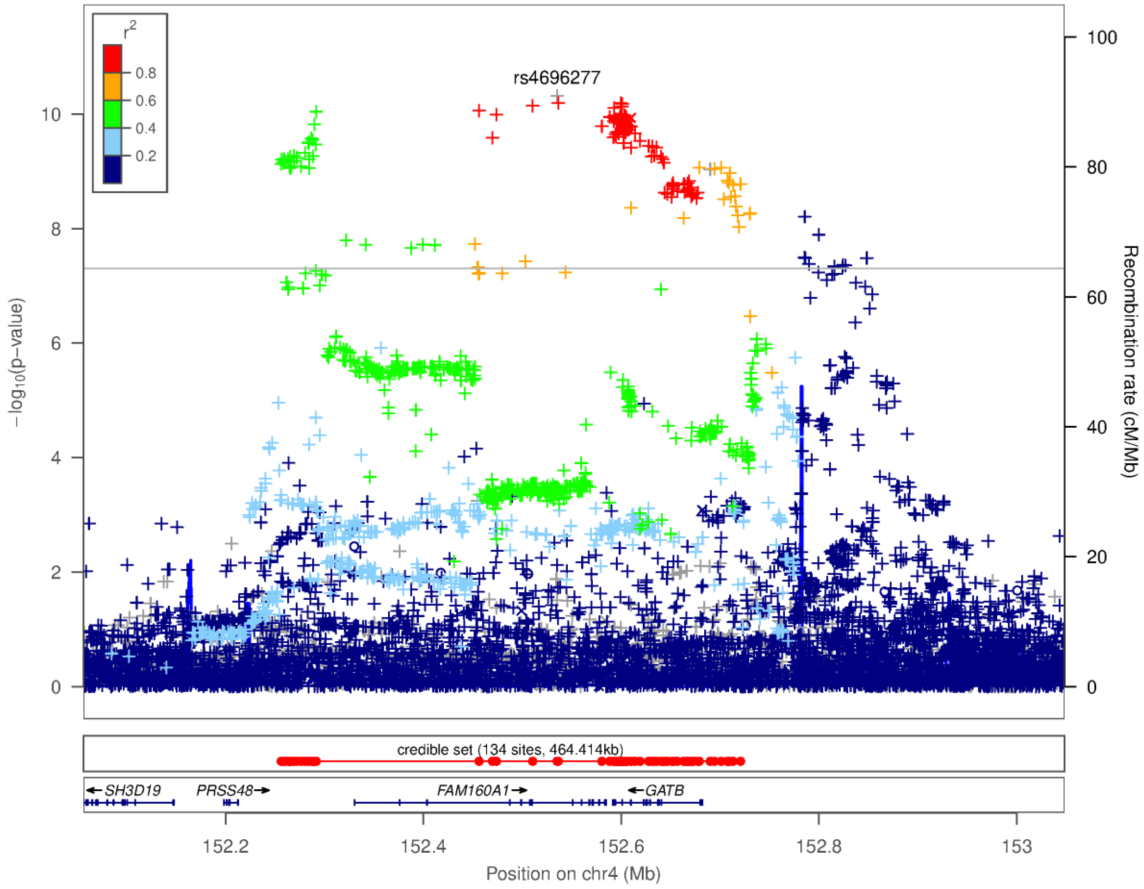

Supplementary Figure 1.xii. Regional association plot which includes information on the credible variant set and known genes in the region for the significant association between dyslexia and chr4q31.3 rs4696277

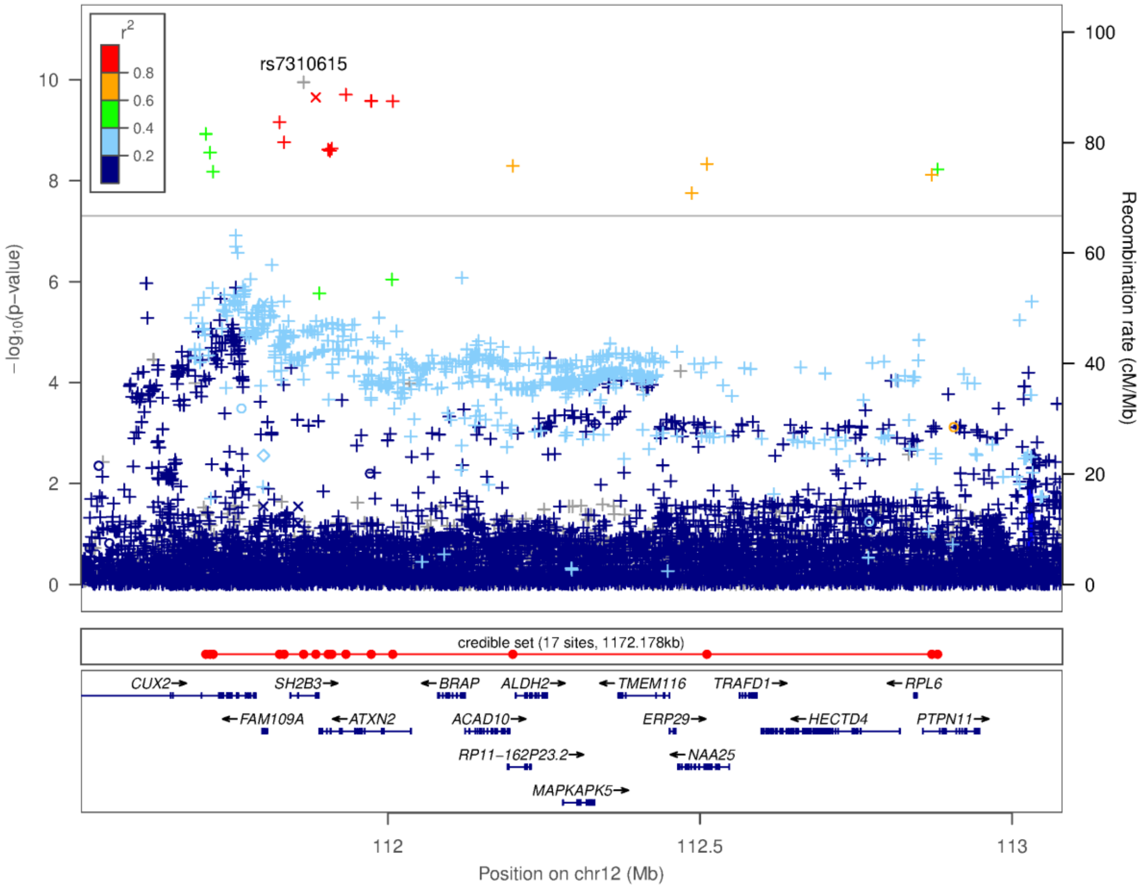

Supplementary Figure 1.xiii. Regional association plot which includes information on the credible variant set and known genes in the region for the significant association between dyslexia and chr12q24.12 rs7310615

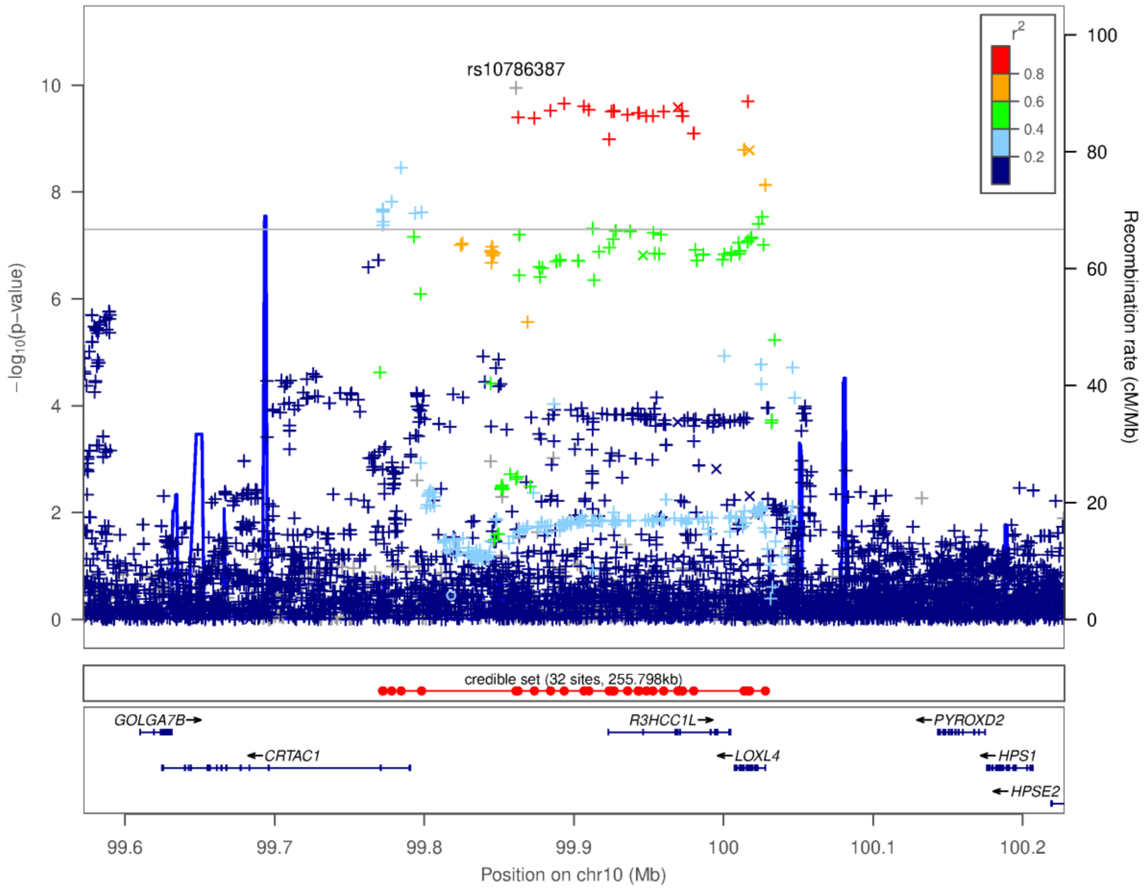

Supplementary Figure 1.xiv. Regional association plot which includes information on the credible variant set and known genes in the region for the significant association between dyslexia and chr10q24.2 rs10786387

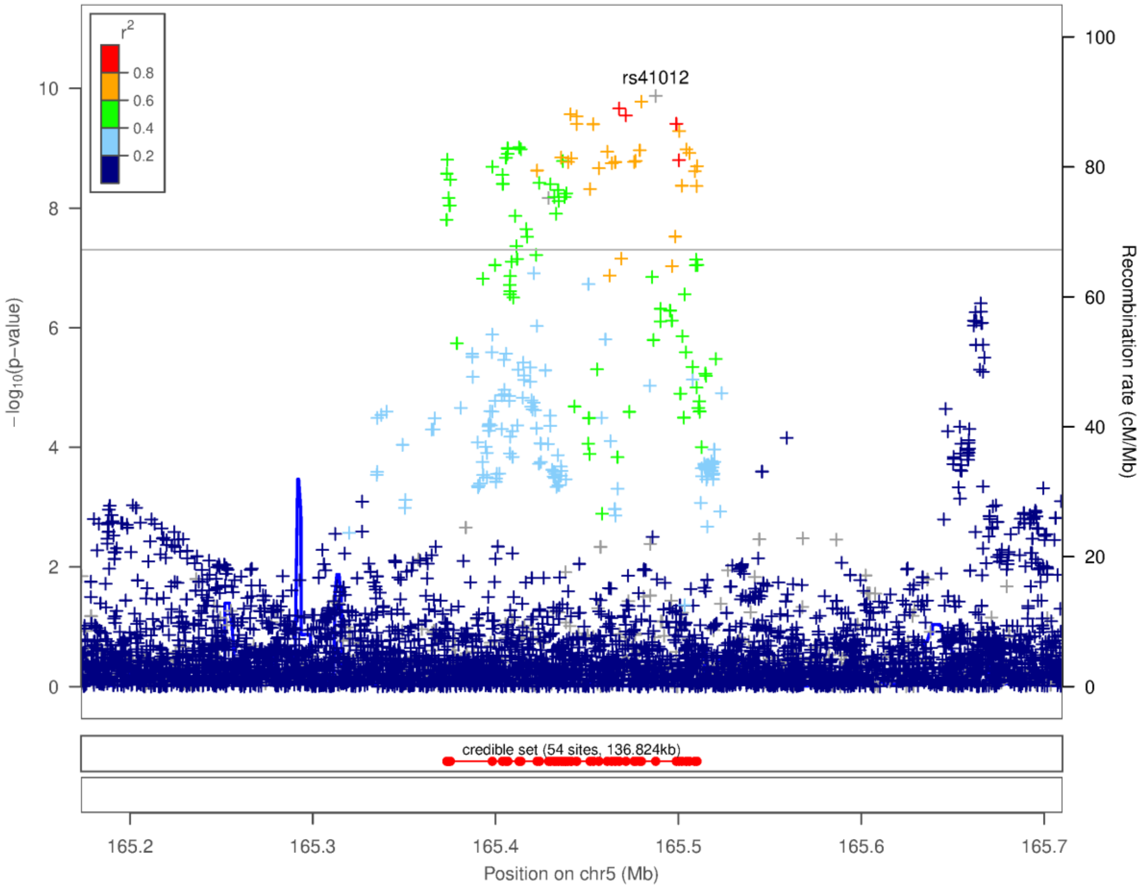

Supplementary Figure 1.xv. Regional association plot which includes information on the credible variant set and known genes in the region for the significant association between dyslexia and chr5q34 rs41012

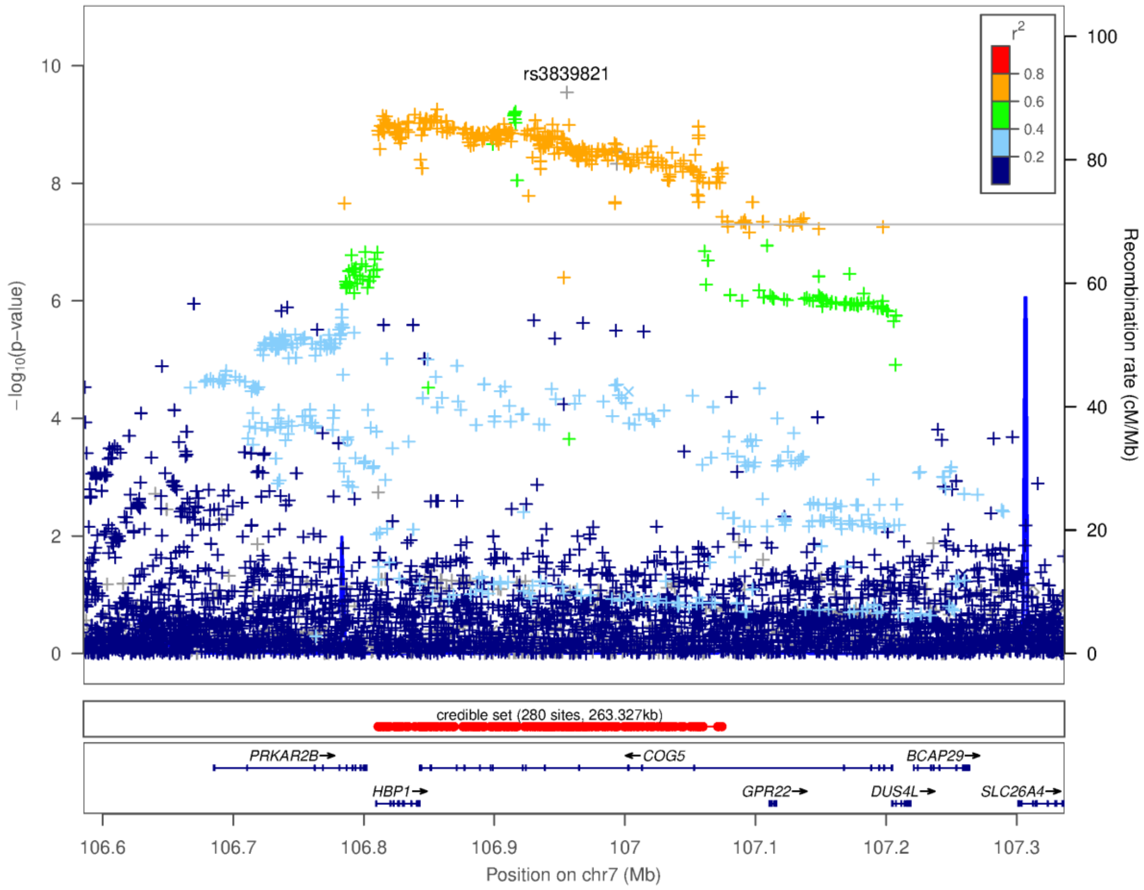

Supplementary Figure 1.xvi. Regional association plot which includes information on the credible variant set and known genes in the region for the significant association between dyslexia and chr7q22.3 rs3839821

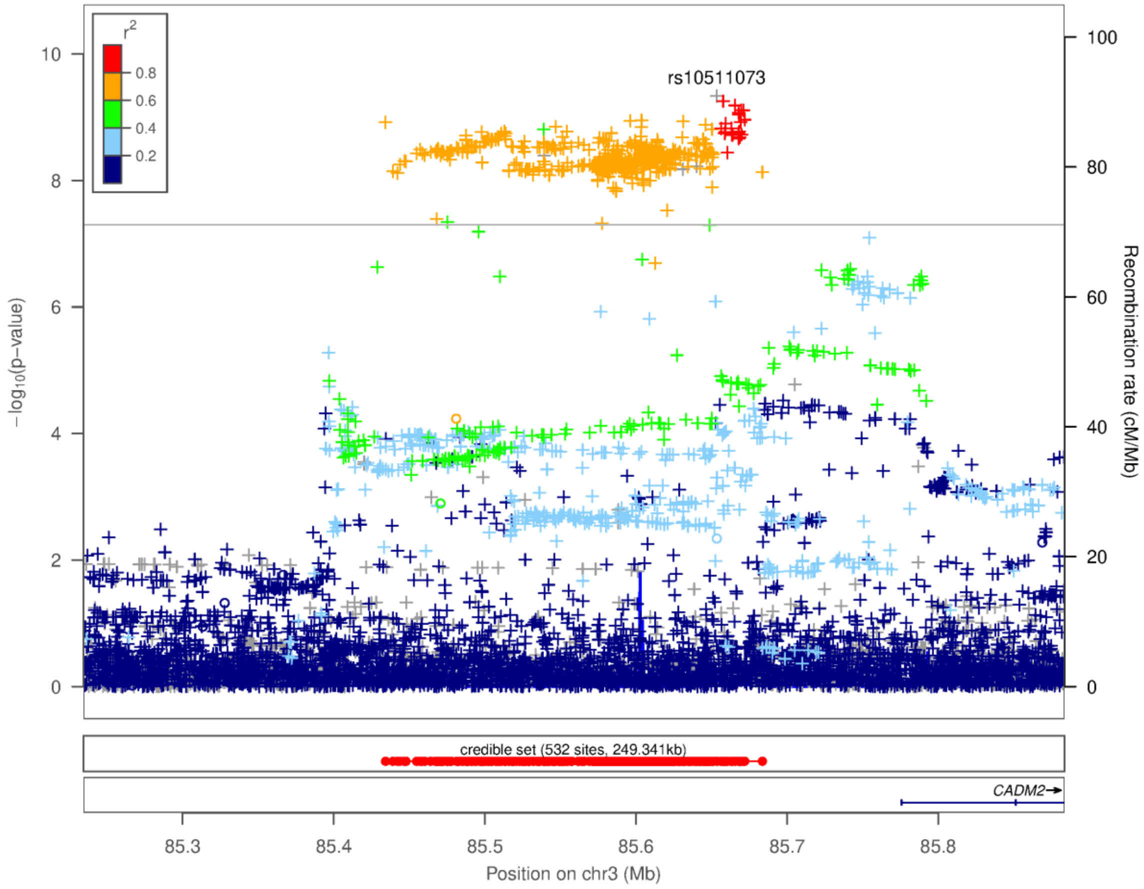

Supplementary Figure 1.xvii. Regional association plot which includes information on the credible variant set and known genes in the region for the significant association between dyslexia and chr3p12.1 rs10511073

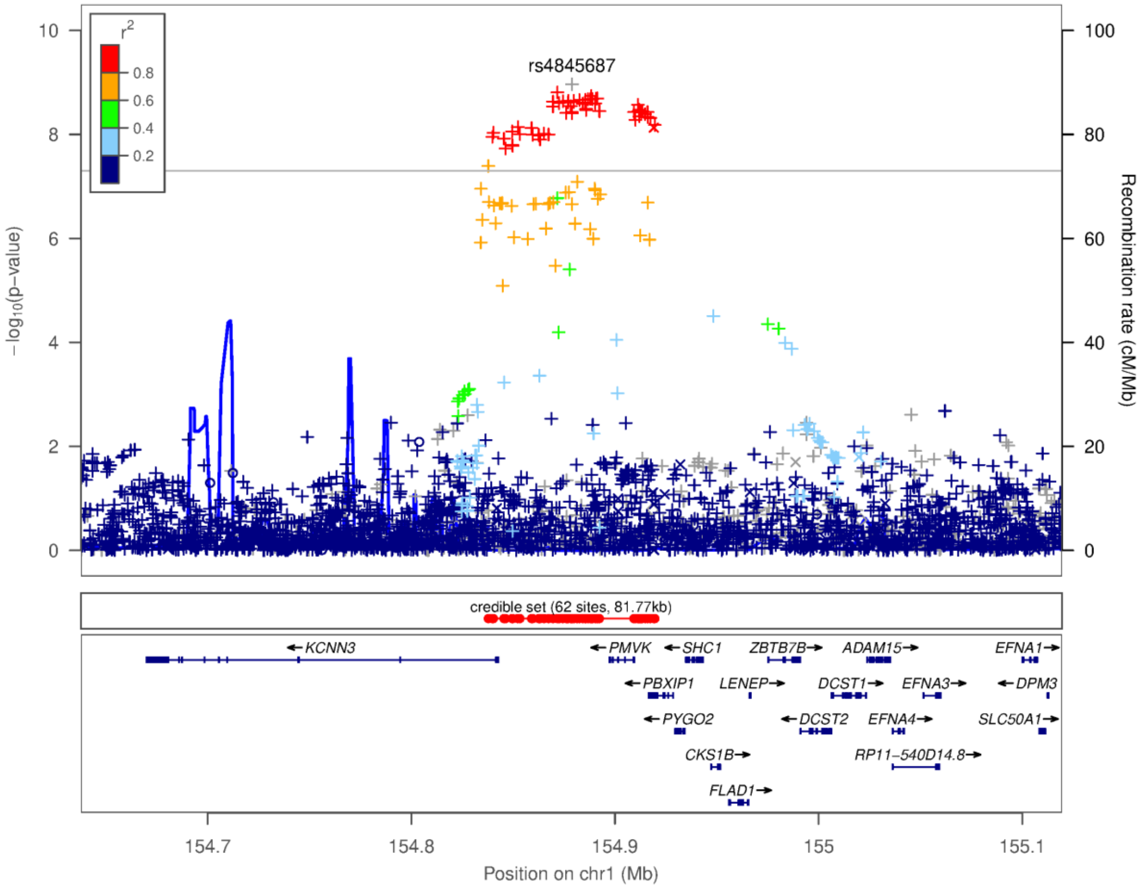

Supplementary Figure 1.xviii. Regional association plot which includes information on the credible variant set and known genes in the region for the significant association between dyslexia and chr1q21.3 rs4845687

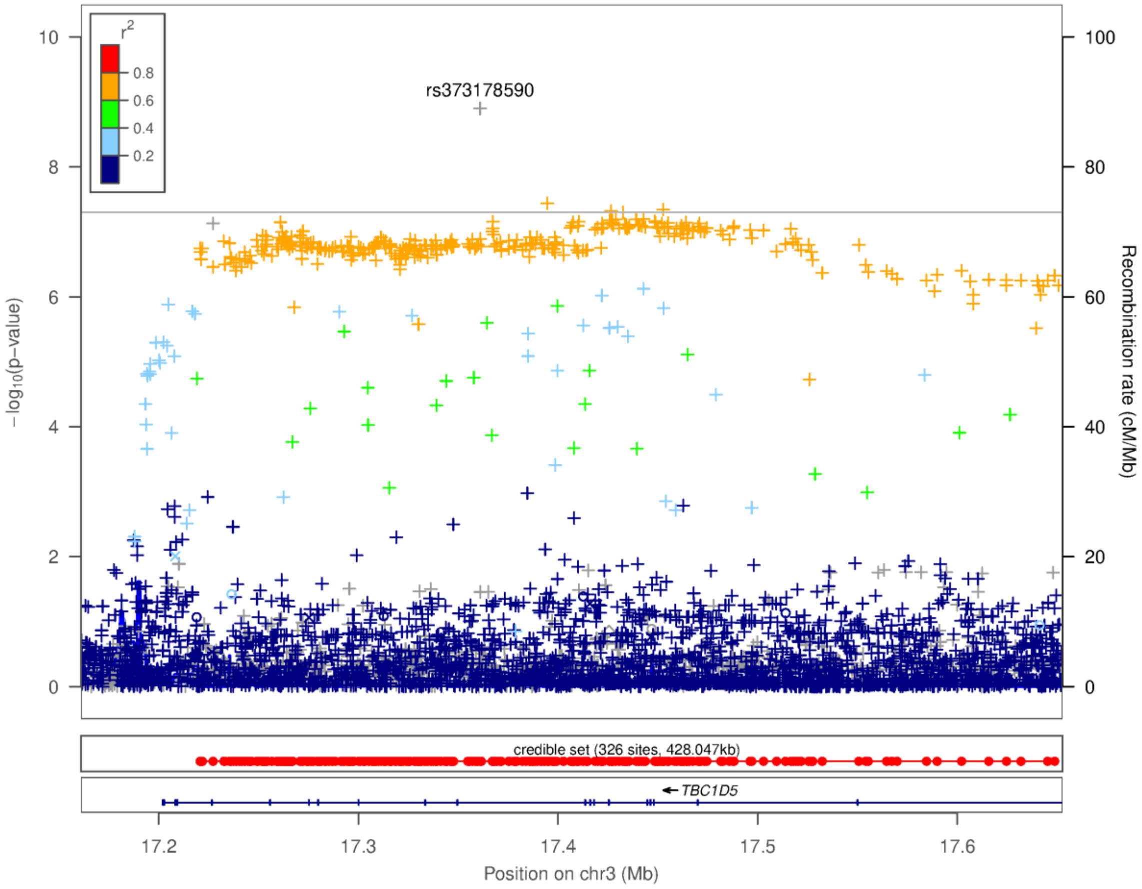

Supplementary Figure 1.xix. Regional association plot which includes information on the credible variant set and known genes in the region for the significant association between dyslexia and chr3p24.3 rs373178590

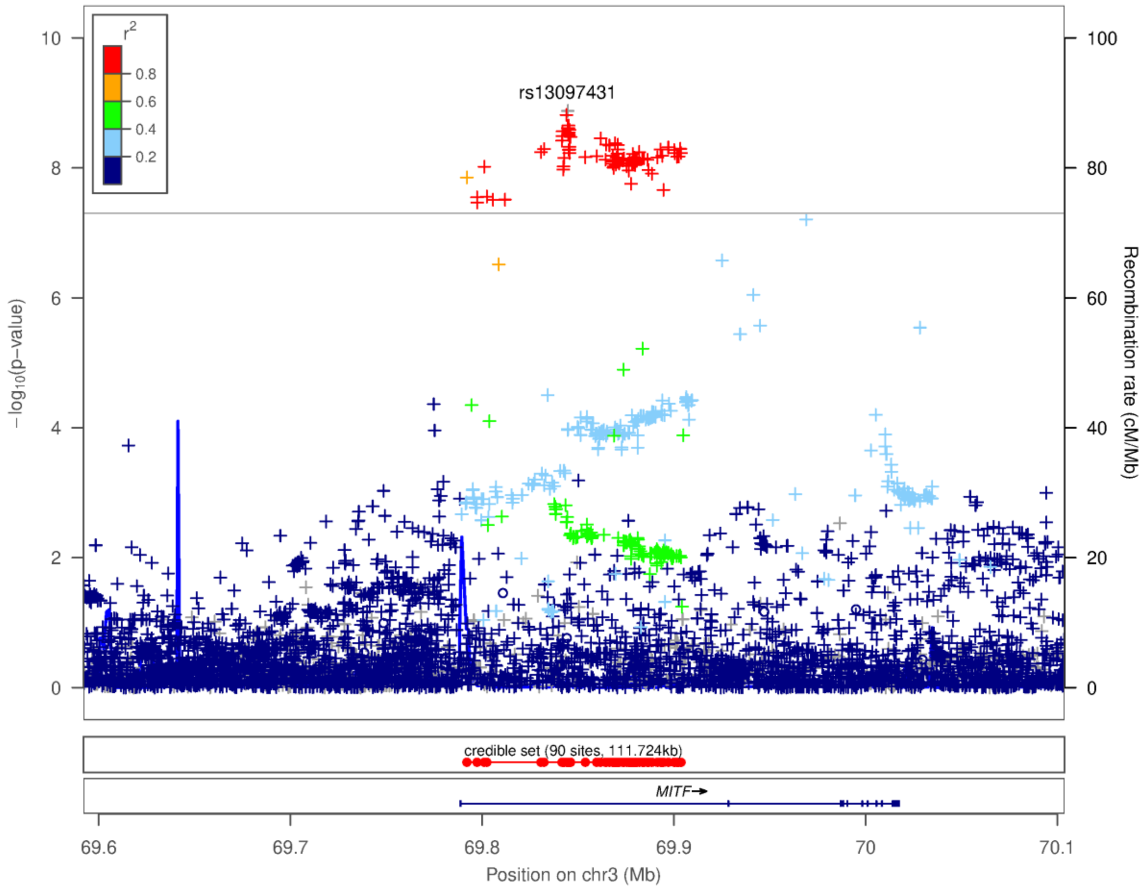

Supplementary Figure 1.xx. Regional association plot which includes information on the credible variant set and known genes in the region for the significant association between dyslexia and chr3p13 rs13097431

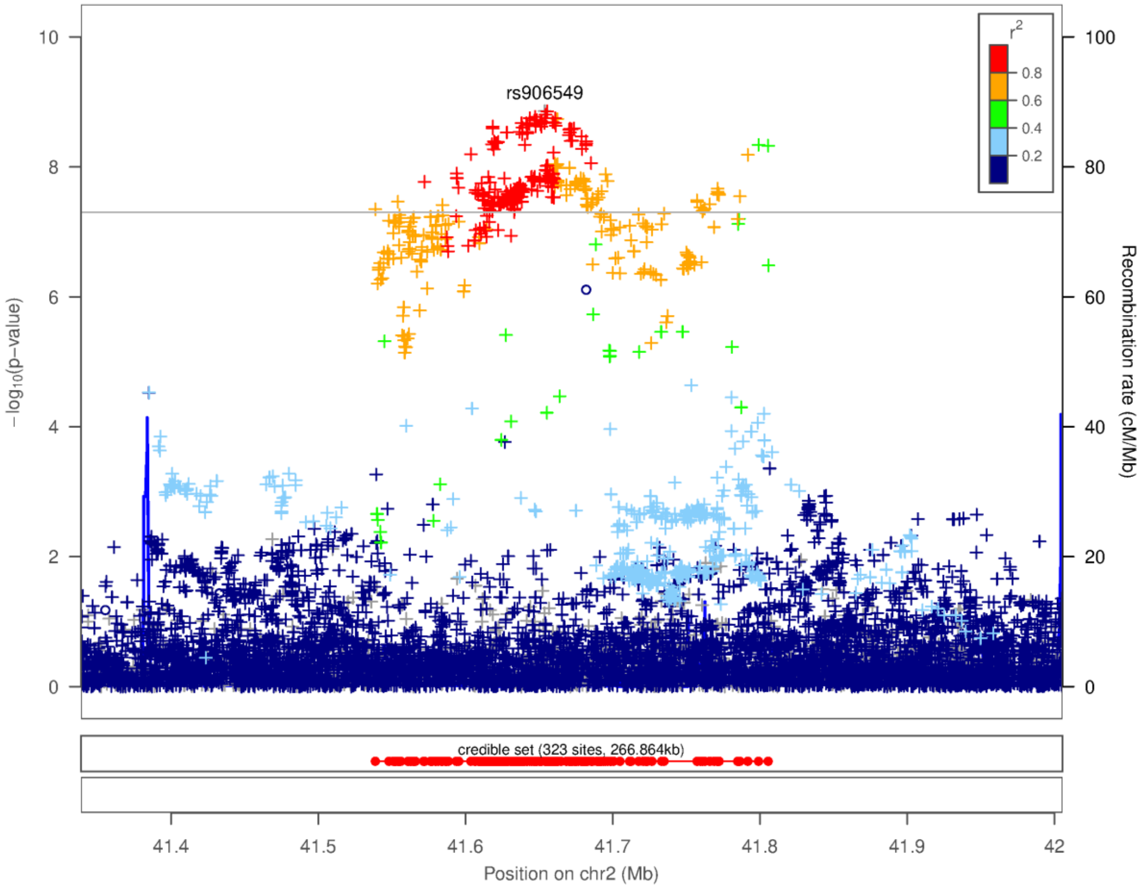

Supplementary Figure 1.xxi. Regional association plot which includes information on the credible variant set and known genes in the region for the significant association between dyslexia and chr2p22.1 rs906549

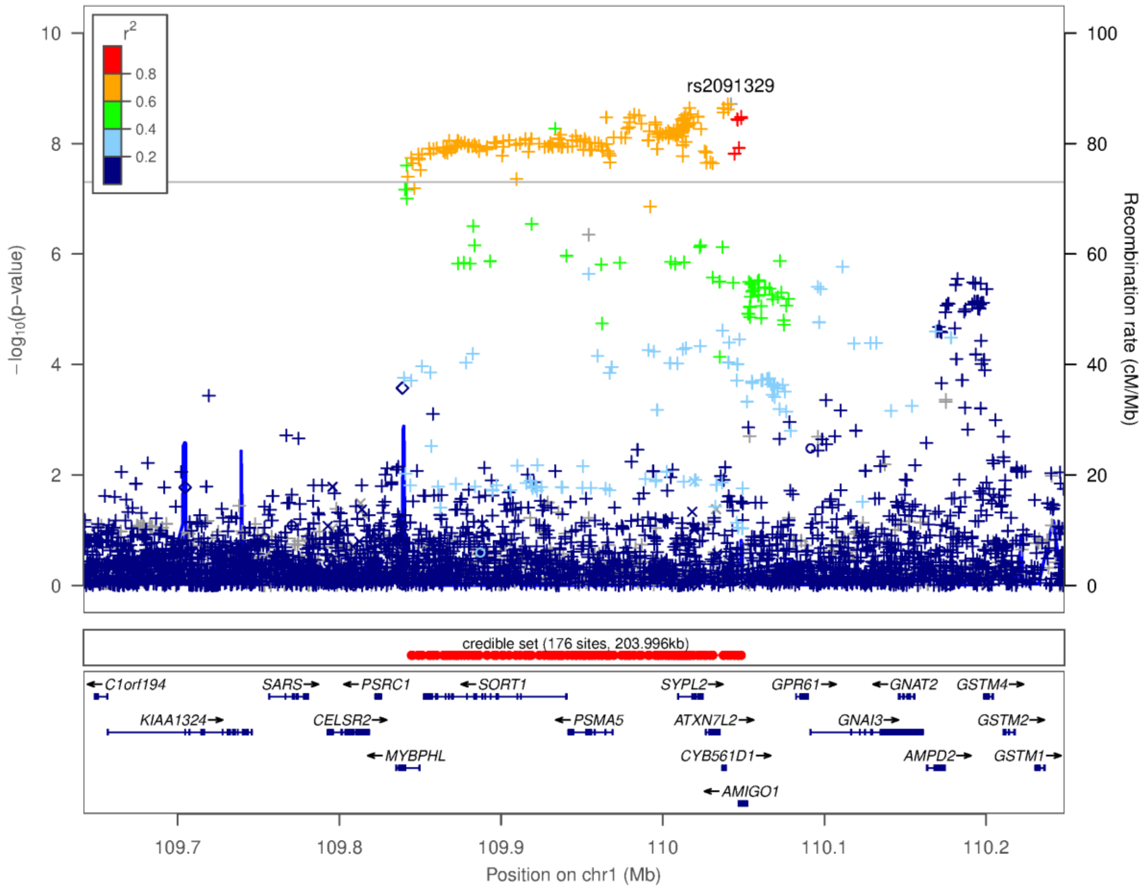

Supplementary Figure 1.xxii. Regional association plot which includes information on the credible variant set and known genes in the region for the significant association between dyslexia and chr1p13.3 rs2091329

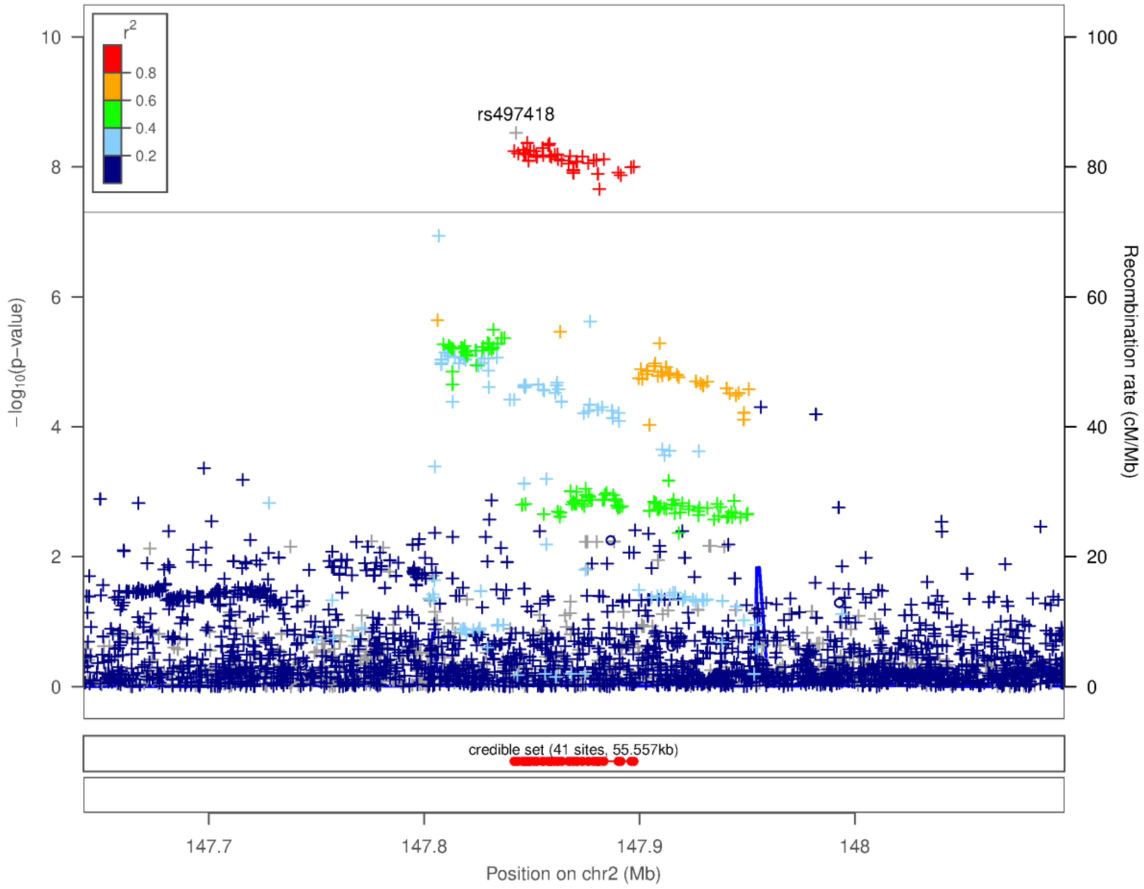

Supplementary Figure 1.xxiii. Regional association plot which includes information on the credible variant set and known genes in the region for the significant association between dyslexia and chr2q22.3 rs497418

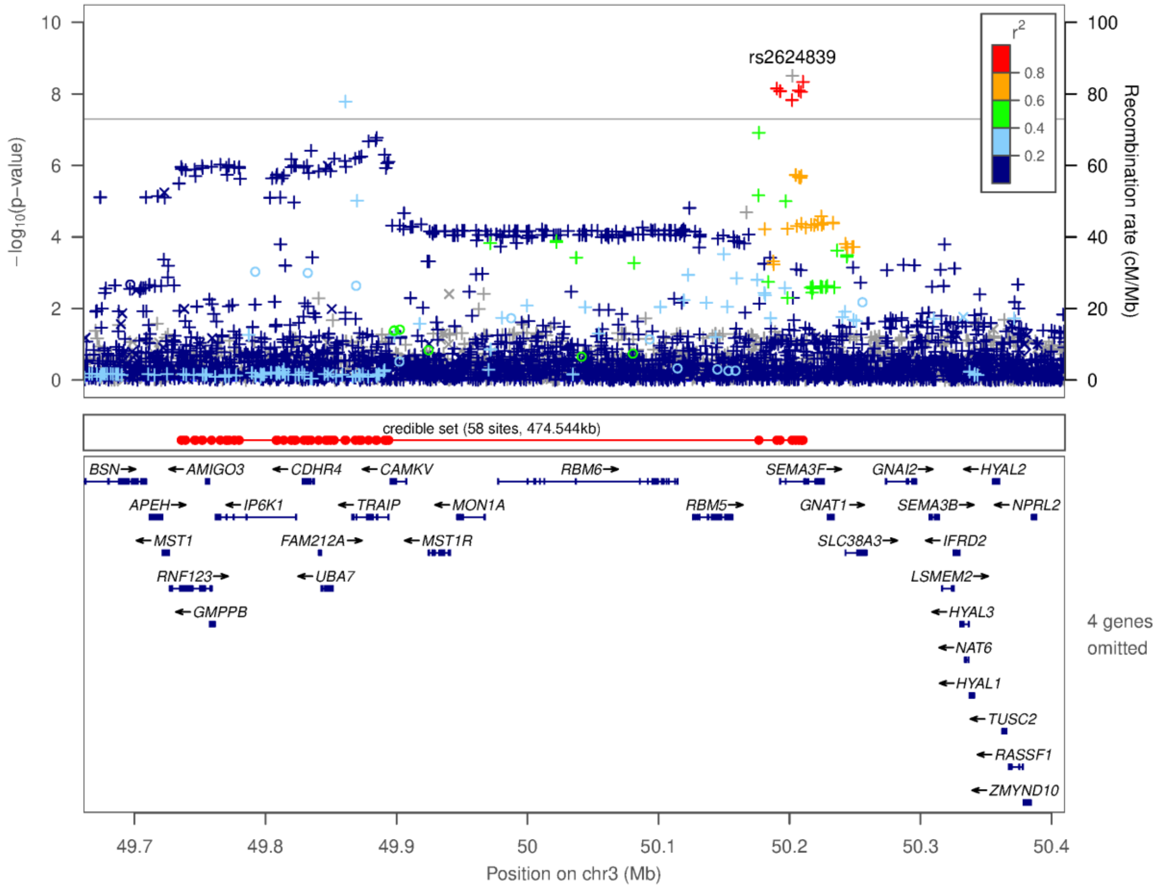

Supplementary Figure 1.xxiv. Regional association plot which includes information on the credible variant set and known genes in the region for the significant association between dyslexia and chr3p21.31 rs2624839

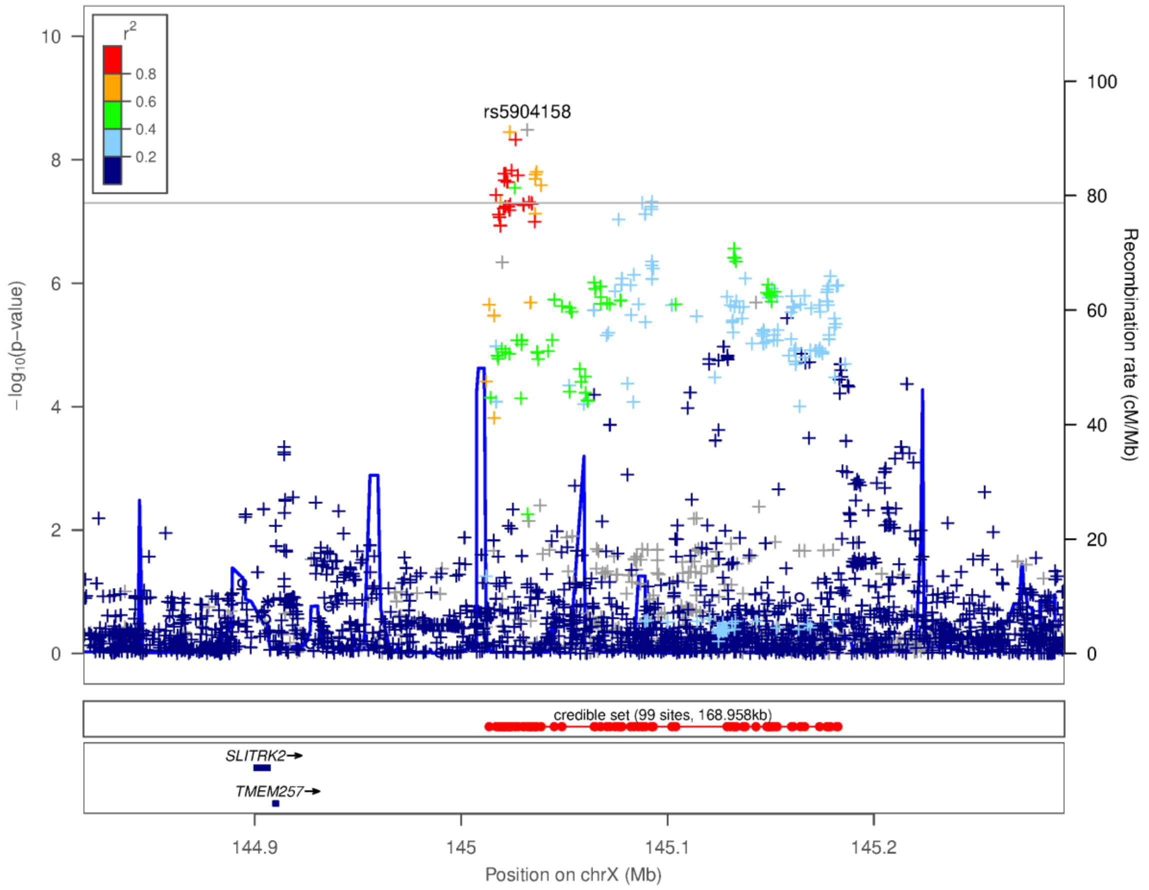

Supplementary Figure 1.xxv. Regional association plot which includes information on the credible variant set and known genes in the region for the significant association between dyslexia and chrXq27.3 rs5904158

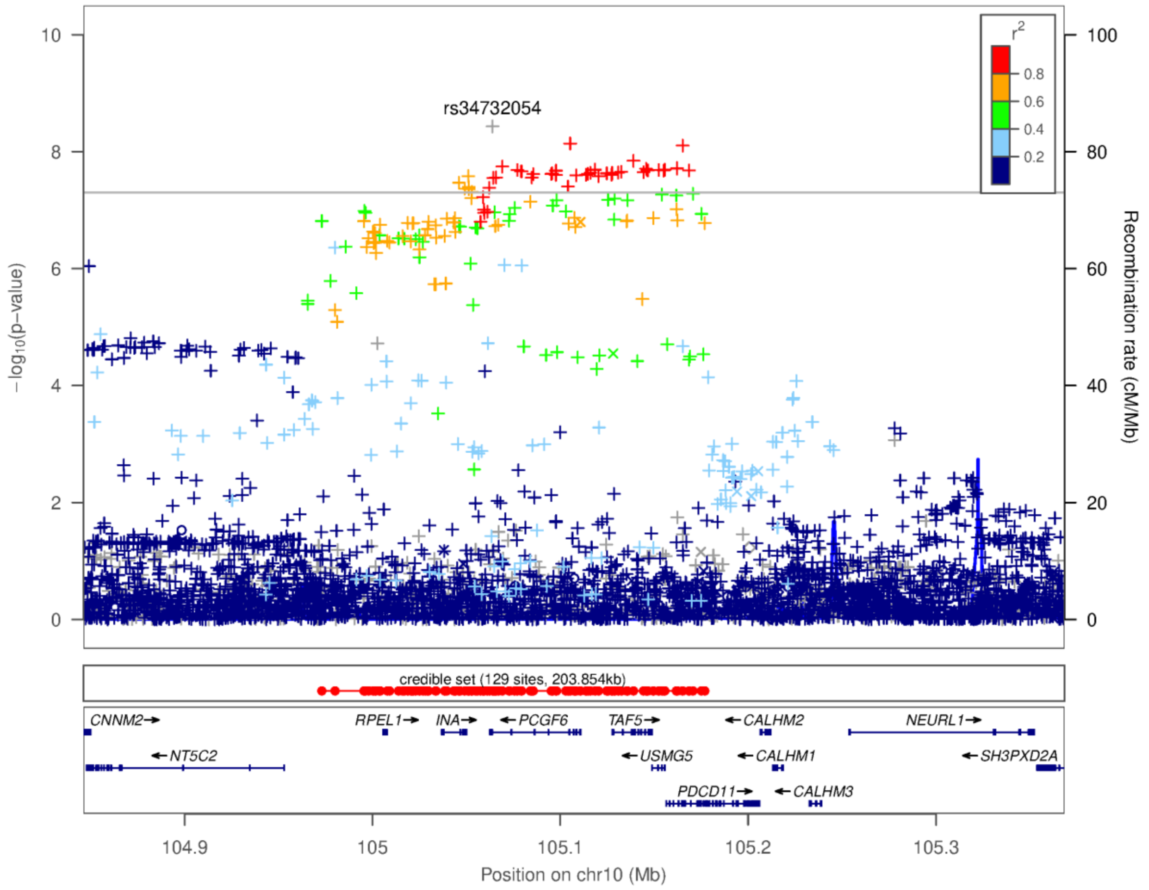

Supplementary Figure 1.xxvi. Regional association plot which includes information on the credible variant set and known genes in the region for the significant association between dyslexia and chr10q24.33 rs34732054

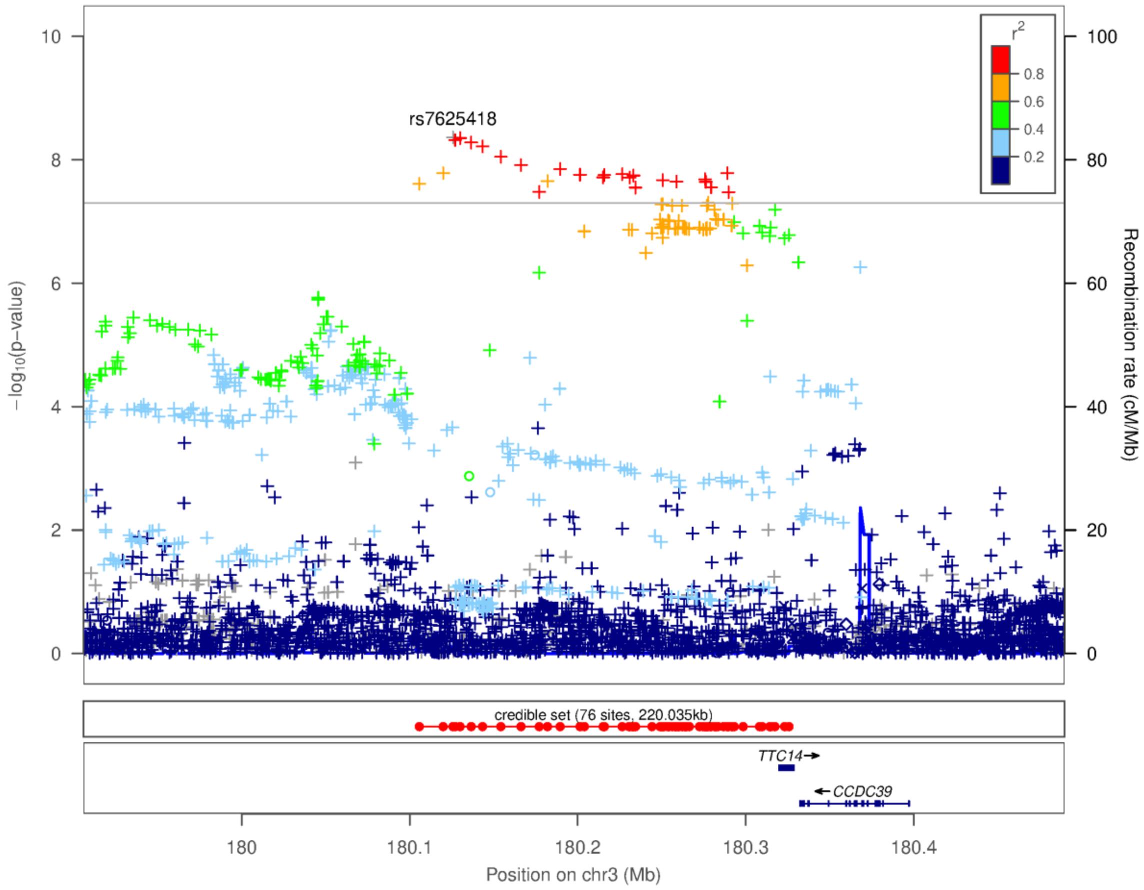

Supplementary Figure 1.xxvii. Regional association plot which includes information on the credible variant set and known genes in the region for the significant association between dyslexia and chr3q26.33 rs7625418

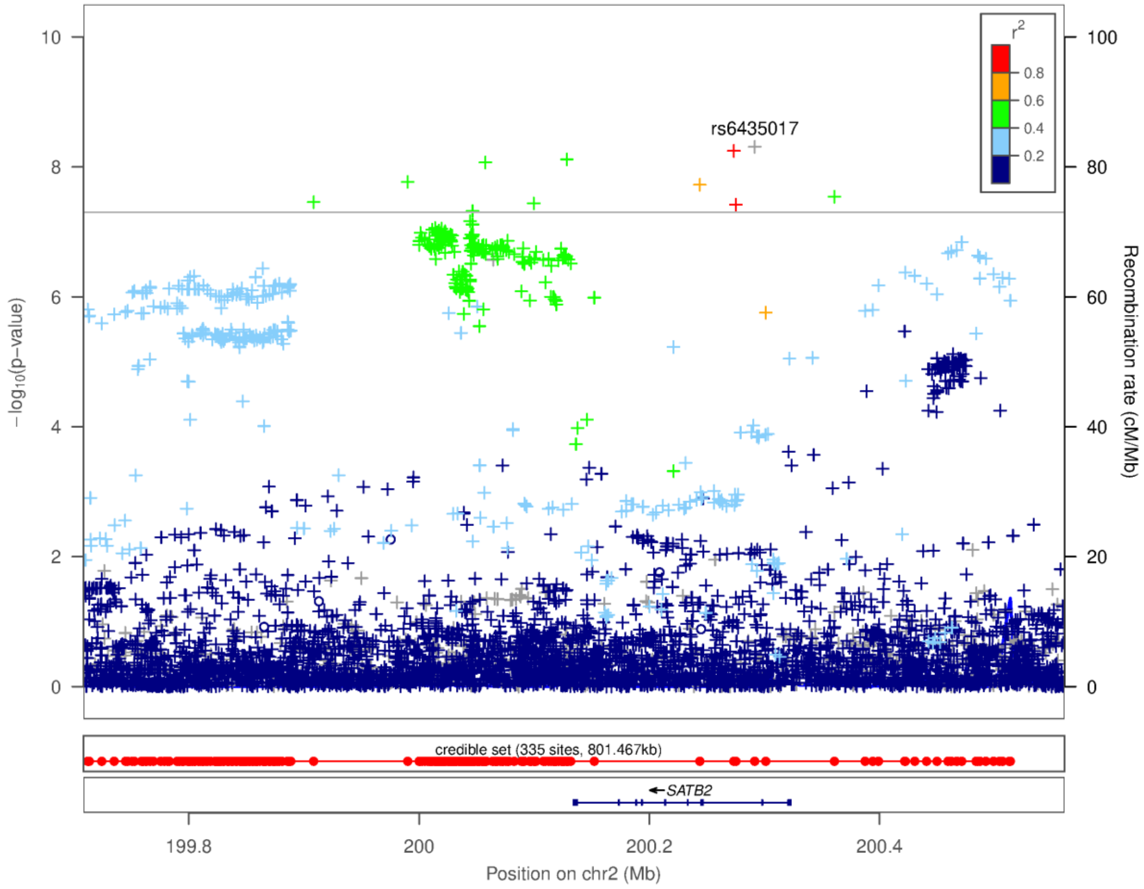

Supplementary Figure 1.xxviii. Regional association plot which includes information on the credible variant set and known genes in the region for the significant association between dyslexia and chr2q33.1 rs6435017

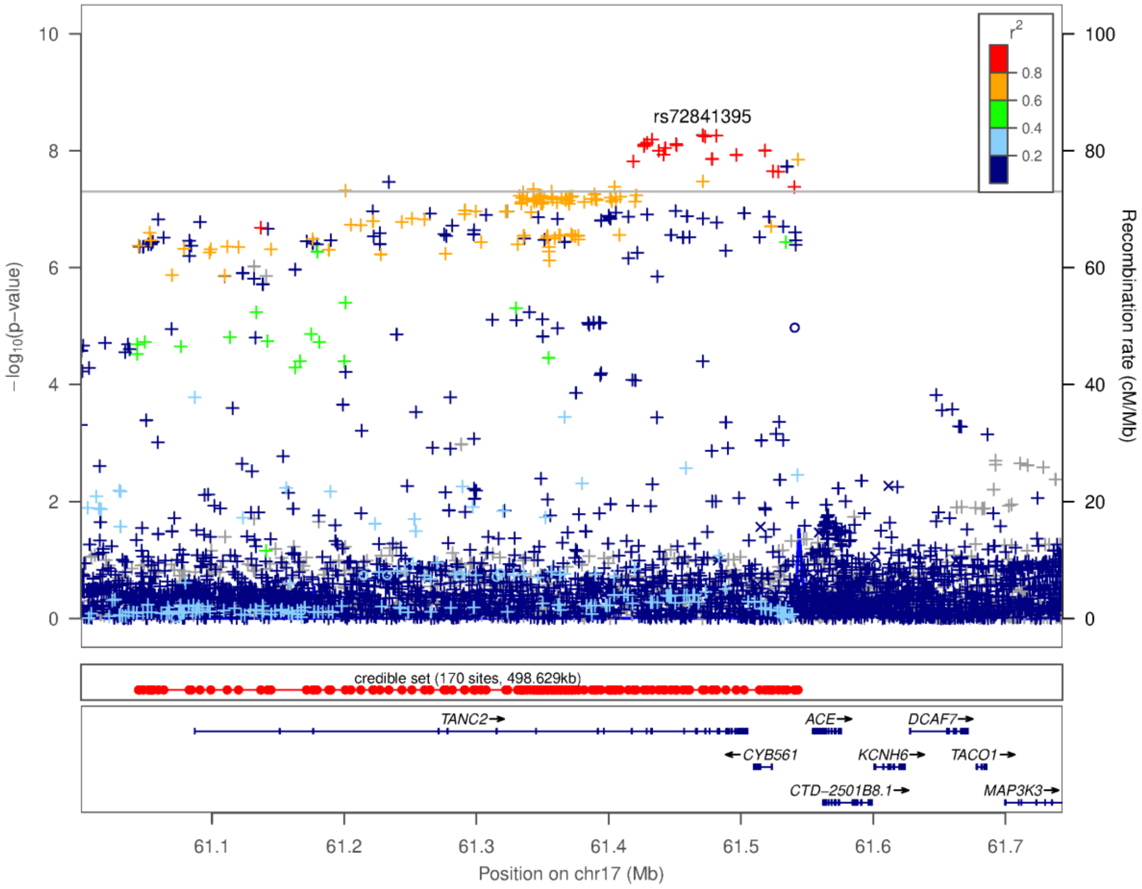

Supplementary Figure 1.xxix. Regional association plot which includes information on the credible variant set and known genes in the region for the significant association between dyslexia and chr17q23.3 rs72841395

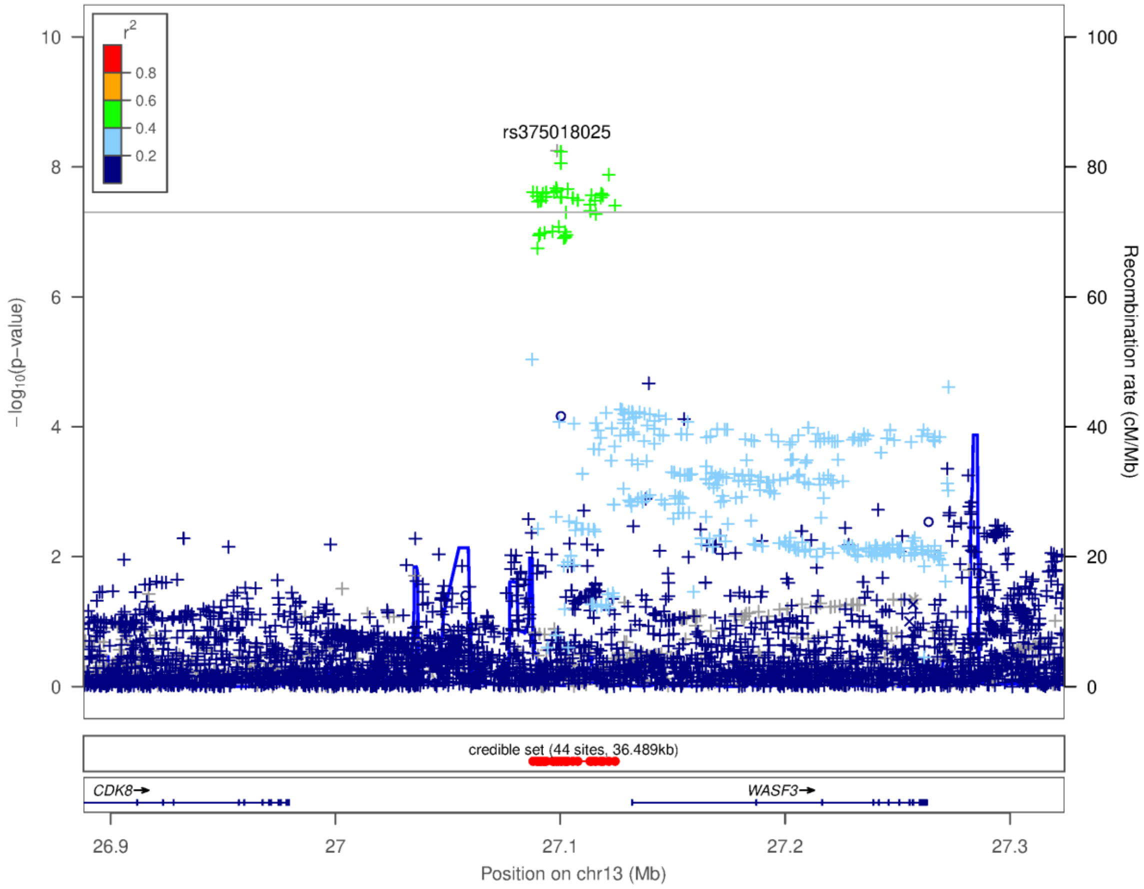

Supplementary Figure 1.xxx. Regional association plot which includes information on the credible variant set and known genes in the region for the significant association between dyslexia and chr13q12.13 rs375018025

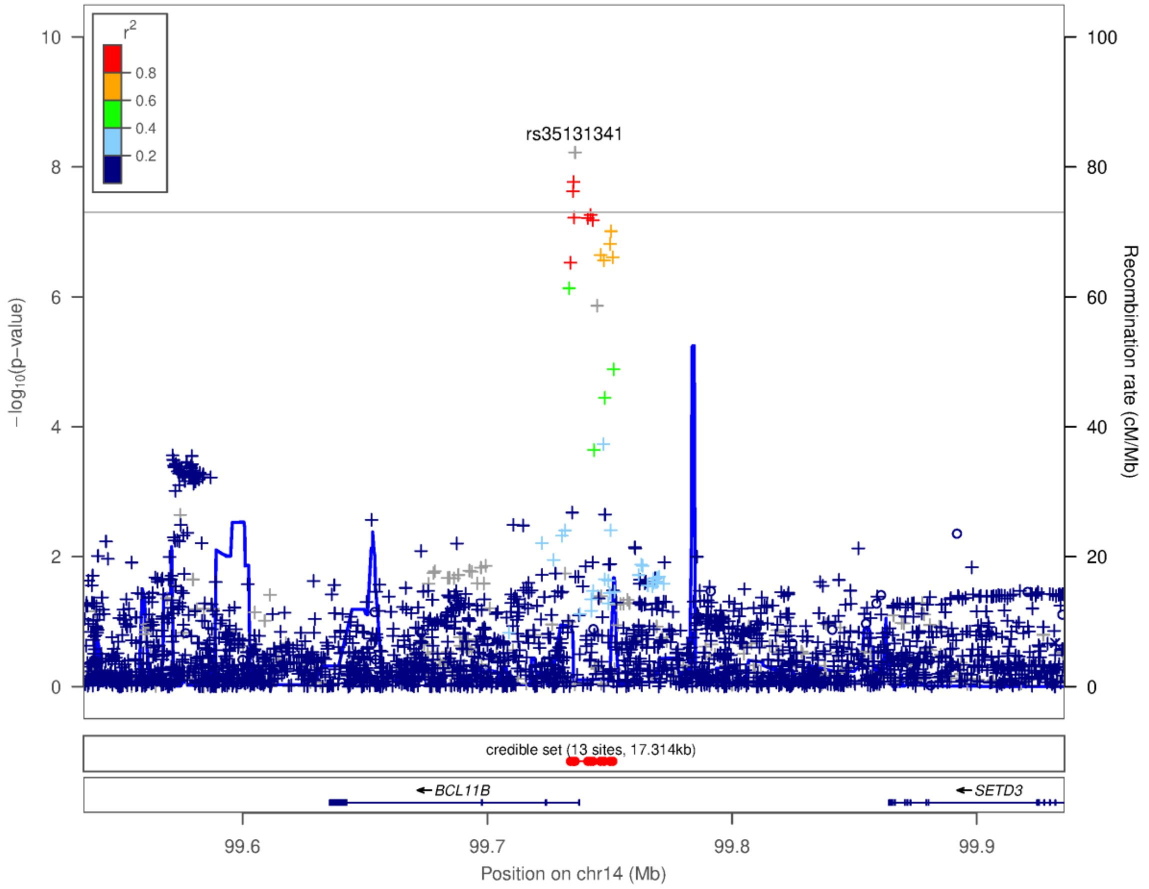

Supplementary Figure 1.xxxi. Regional association plot which includes information on the credible variant set and known genes in the region for the significant association between dyslexia and chr14q32.2 rs35131341

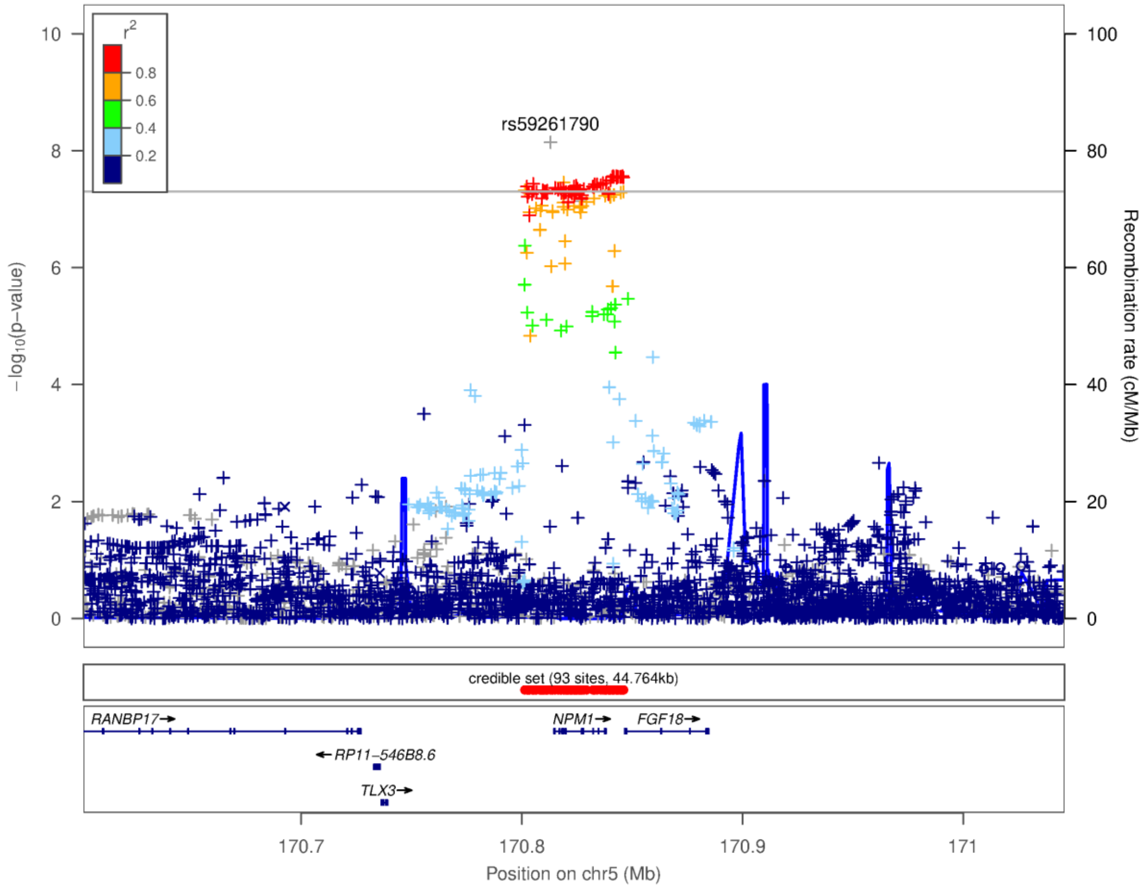

Supplementary Figure 1.xxxii. Regional association plot which includes information on the credible variant set and known genes in the region for the significant association between dyslexia and chr5q35.1 rs59261790

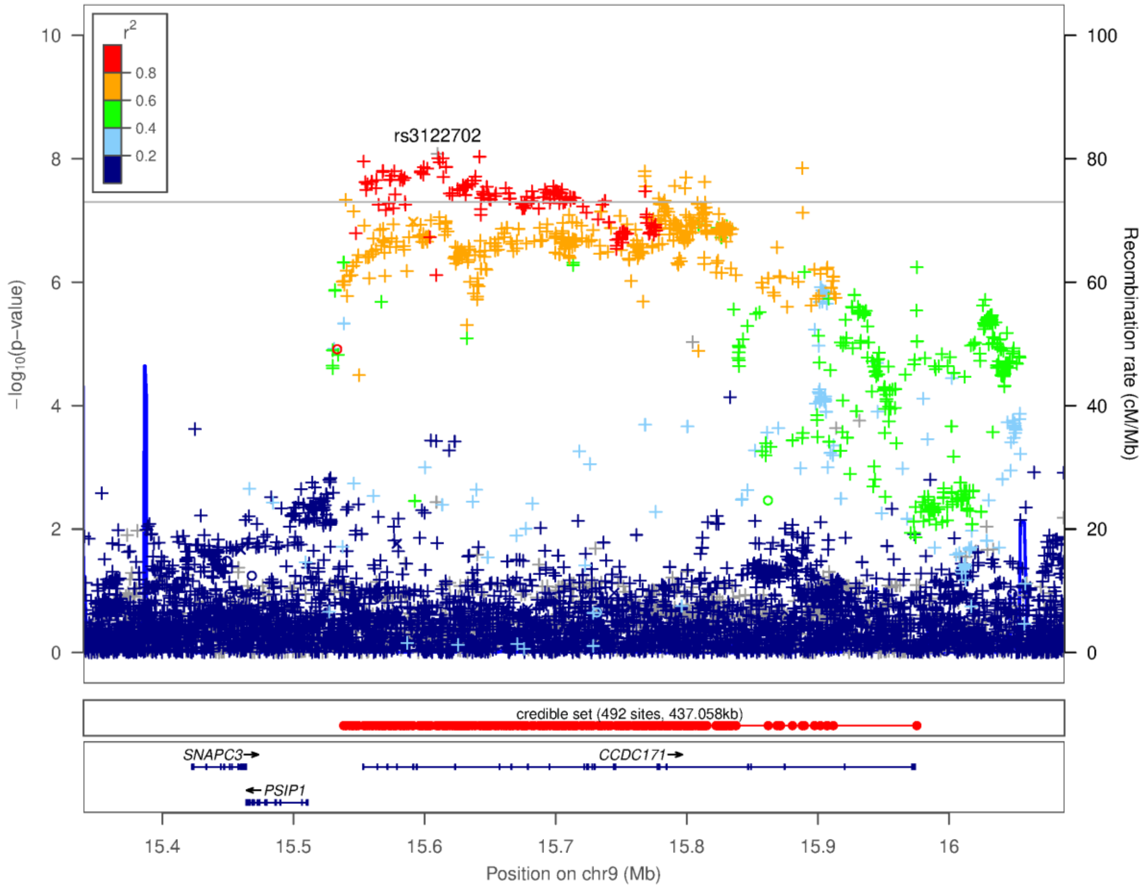

Supplementary Figure 1.xxxiii. Regional association plot which includes information on the credible variant set and known genes in the region for the significant association between dyslexia and chr9p22.3 rs3122702

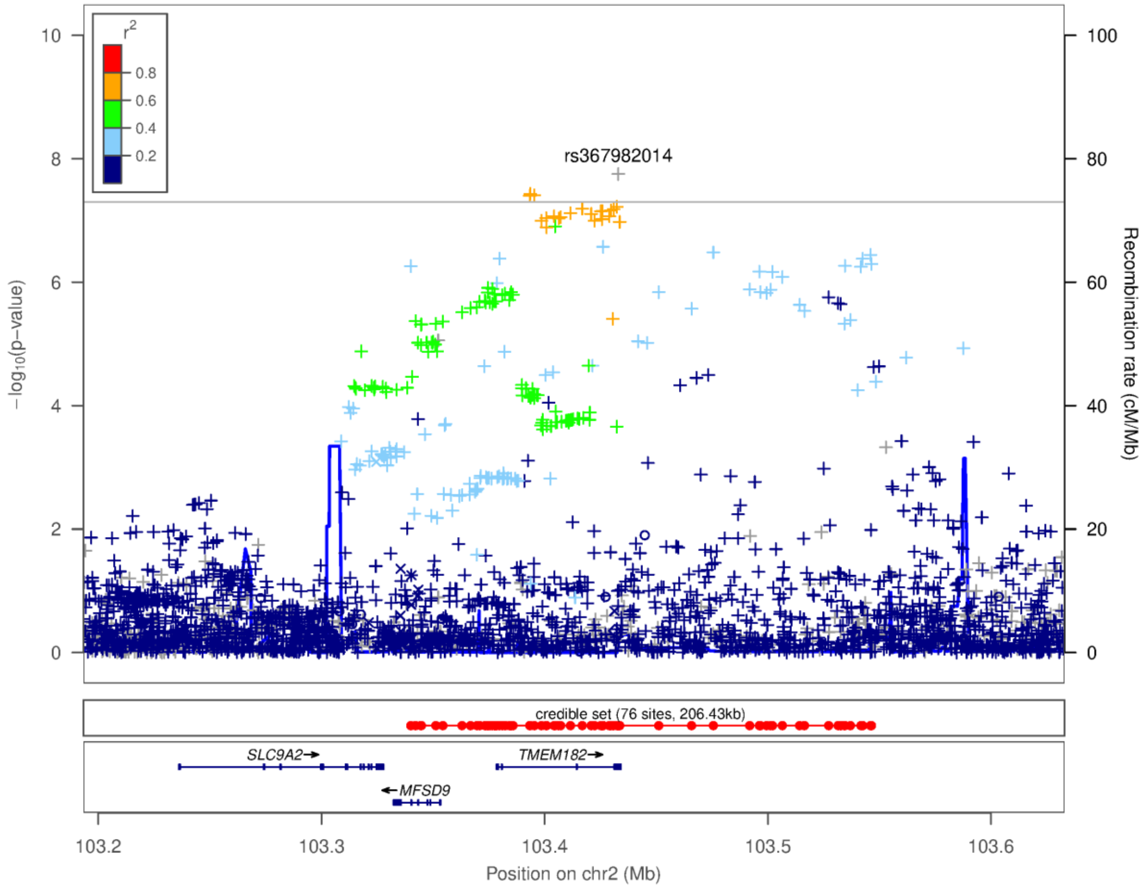

Supplementary Figure 1.xxxiv. Regional association plot which includes information on the credible variant set and known genes in the region for the significant association between dyslexia and chr2q12.1 rs367982014

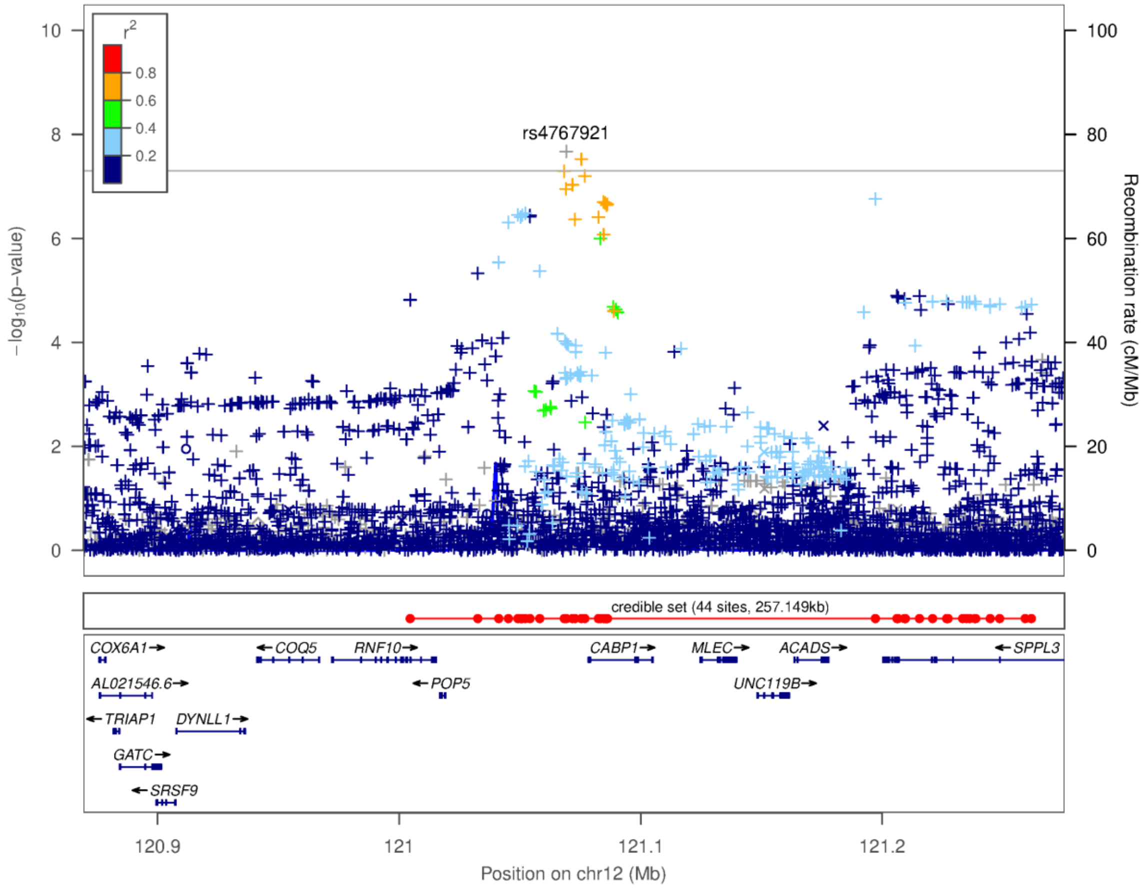

Supplementary Figure 1.xxxv. Regional association plot which includes information on the credible variant set and known genes in the region for the significant association between dyslexia and chr12q24.31 rs4767921

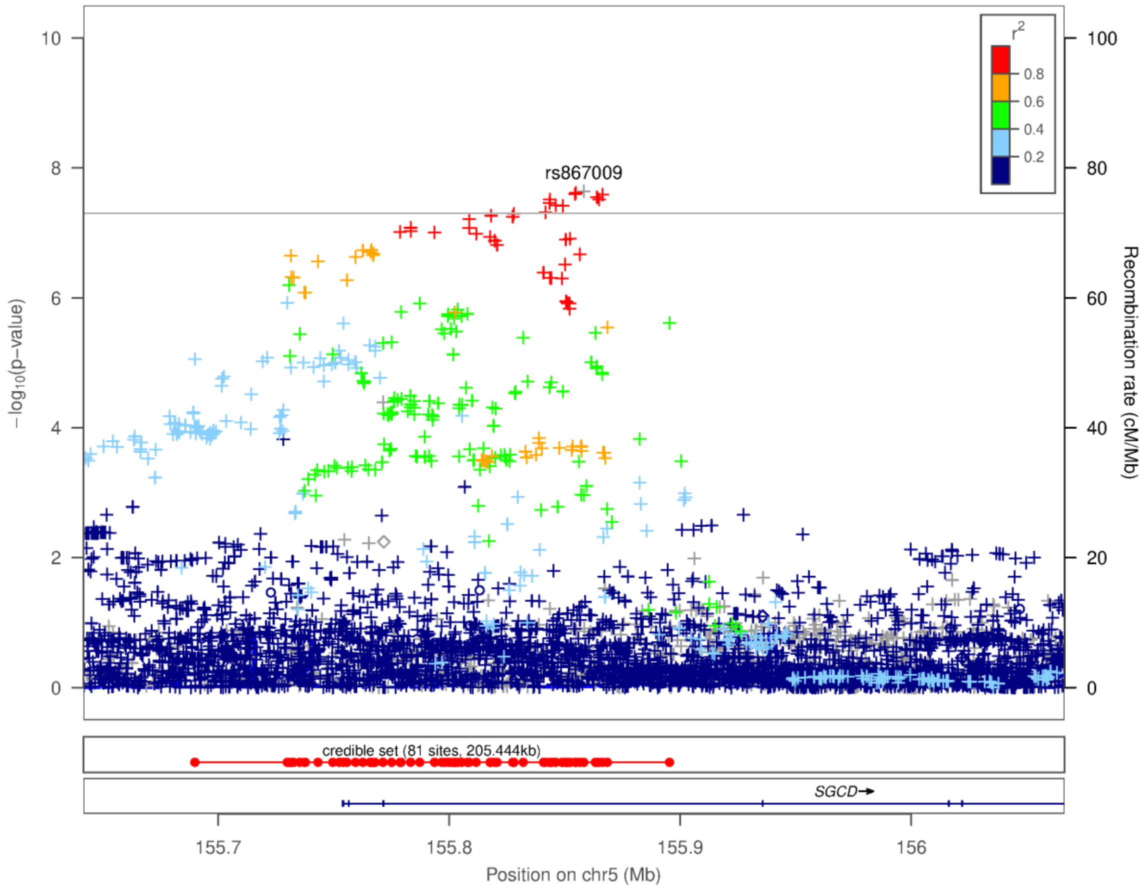

Supplementary Figure 1.xxxvi. Regional association plot which includes information on the credible variant set and known genes in the region for the significant association between dyslexia and chr5q33.3 rs867009

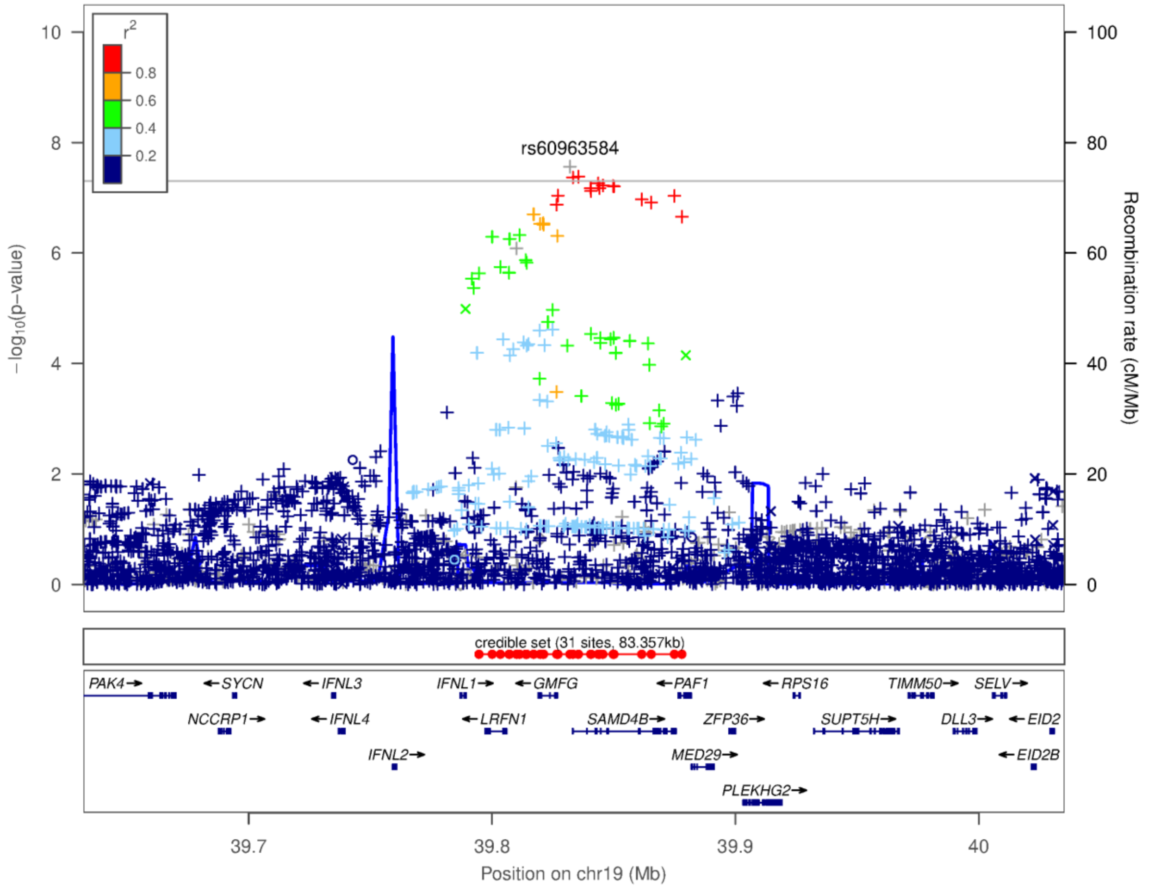

Supplementary Figure 1.xxxvii. Regional association plot which includes information on the credible variant set and known genes in the region for the significant association between dyslexia and chr19q13.2 rs60963584

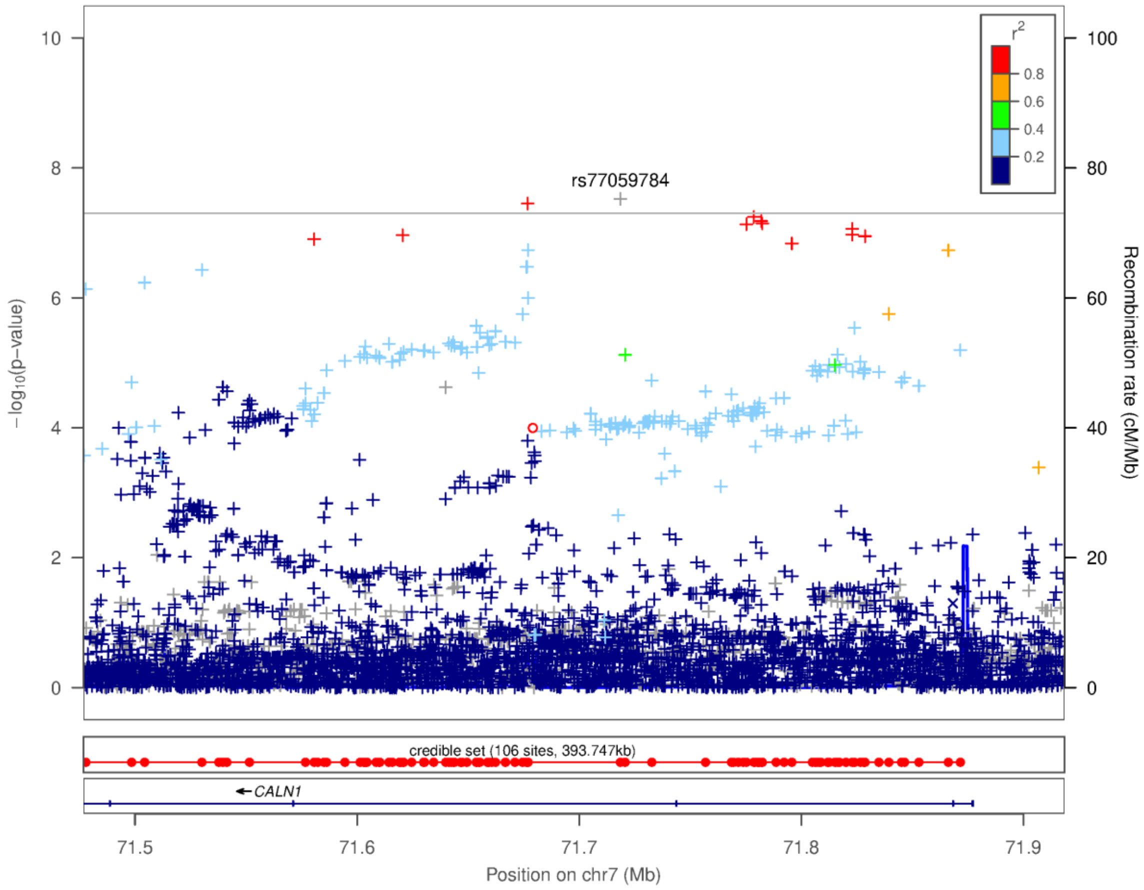

Supplementary Figure 1.xxxviii. Regional association plot which includes information on the credible variant set and known genes in the region for the significant association between dyslexia and chr7q11.22 rs77059784

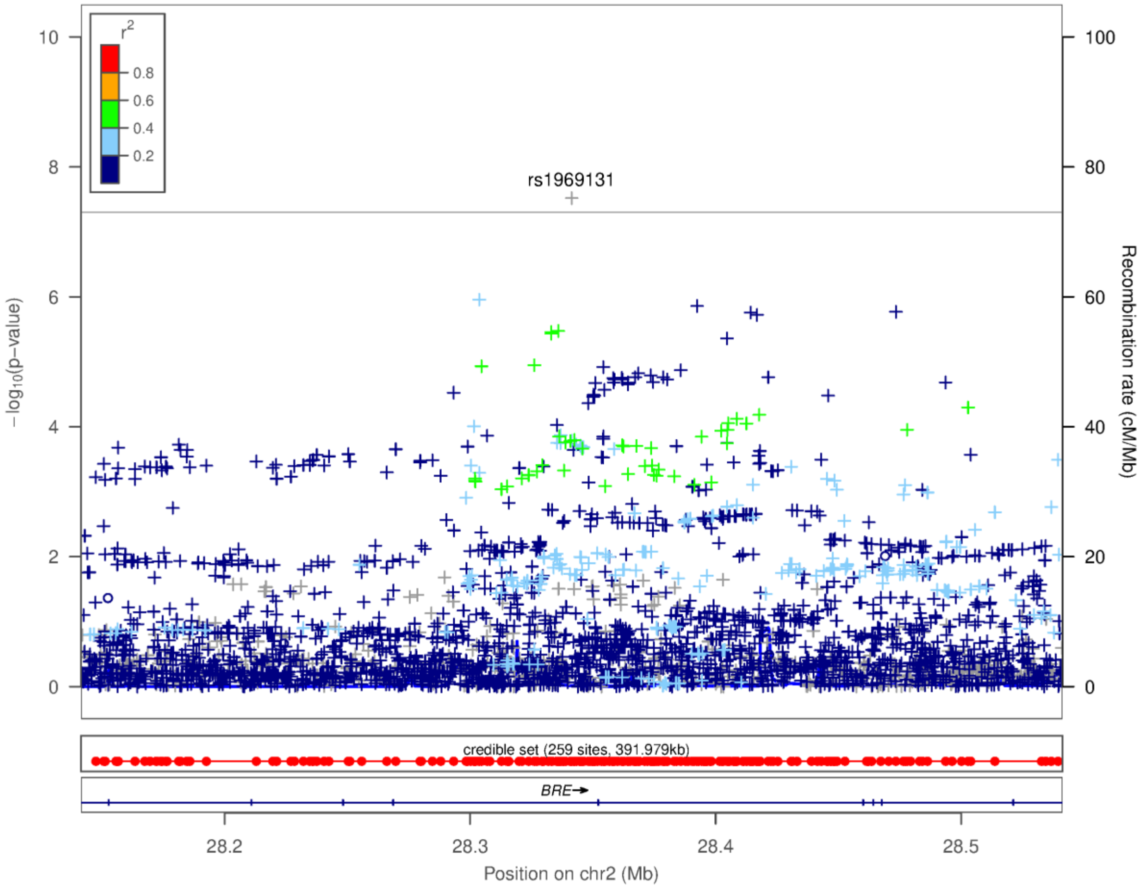

Supplementary Figure 1.xxxix. Regional association plot which includes information on the credible variant set and known genes in the region for the significant association between dyslexia and chr2p23.2 rs1969131

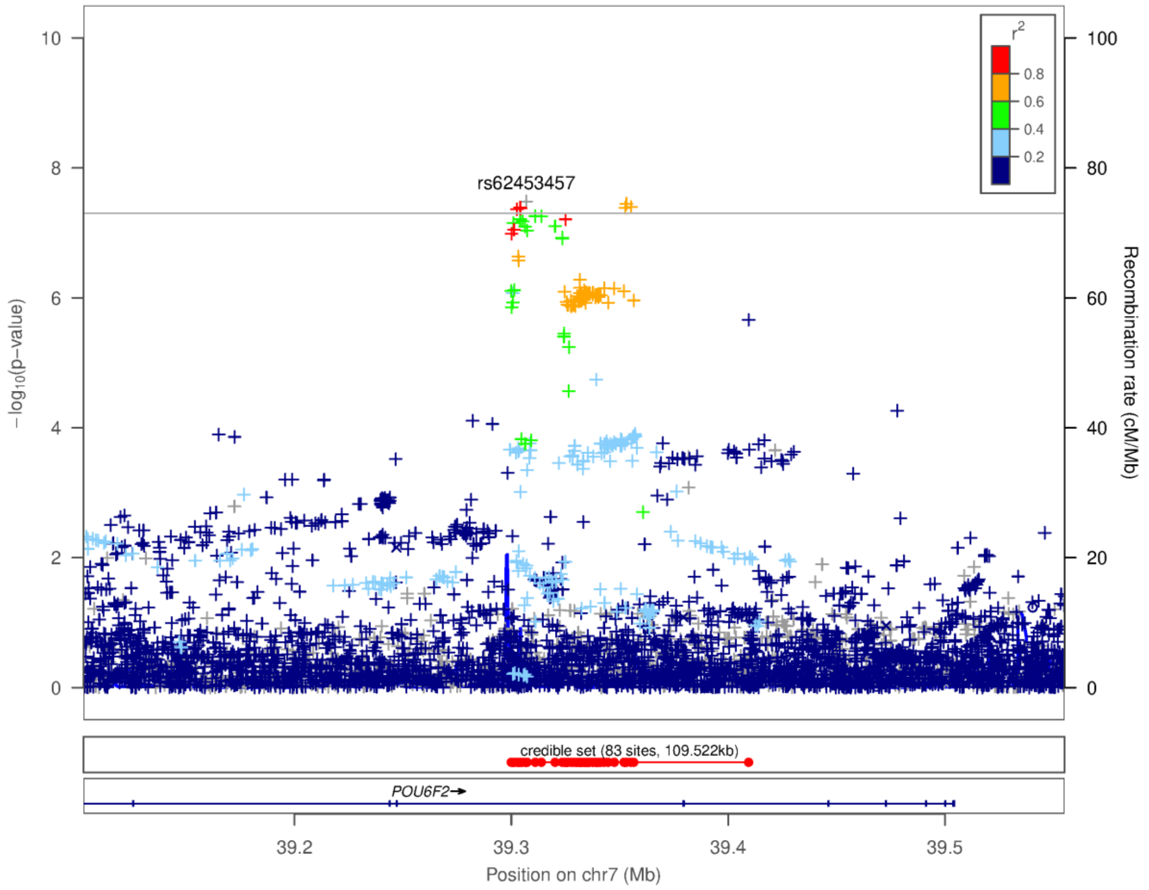

Supplementary Figure 1.xl. Regional association plot which includes information on the credible variant set and known genes in the region for the significant association between dyslexia and chr7p14.1 rs62453457

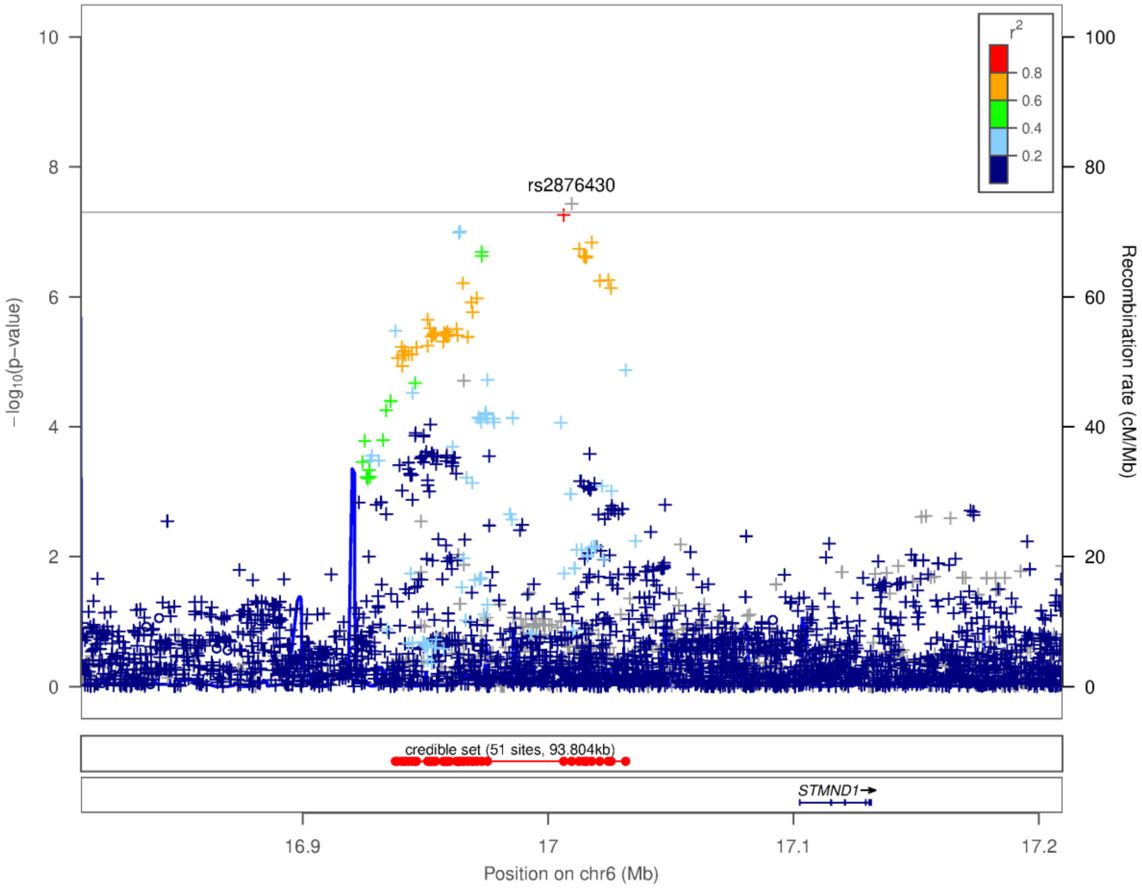

Supplementary Figure 1.xli. Regional association plot which includes information on the credible variant set and known genes in the region for the significant association between dyslexia and chr6p22.3 rs2876430

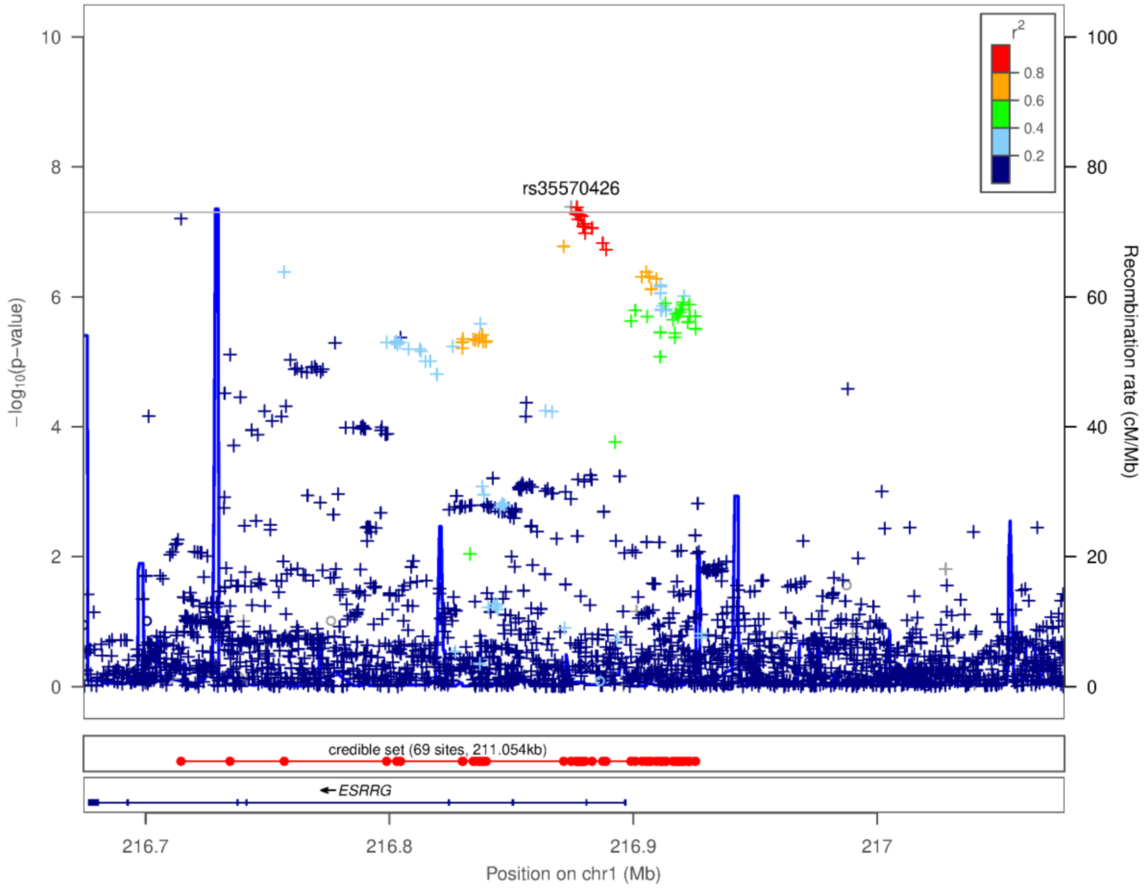

Supplementary Figure 1.xlii. Regional association plot which includes information on the credible variant set and known genes in the region for the significant association between dyslexia and chr1q41 rs35570426

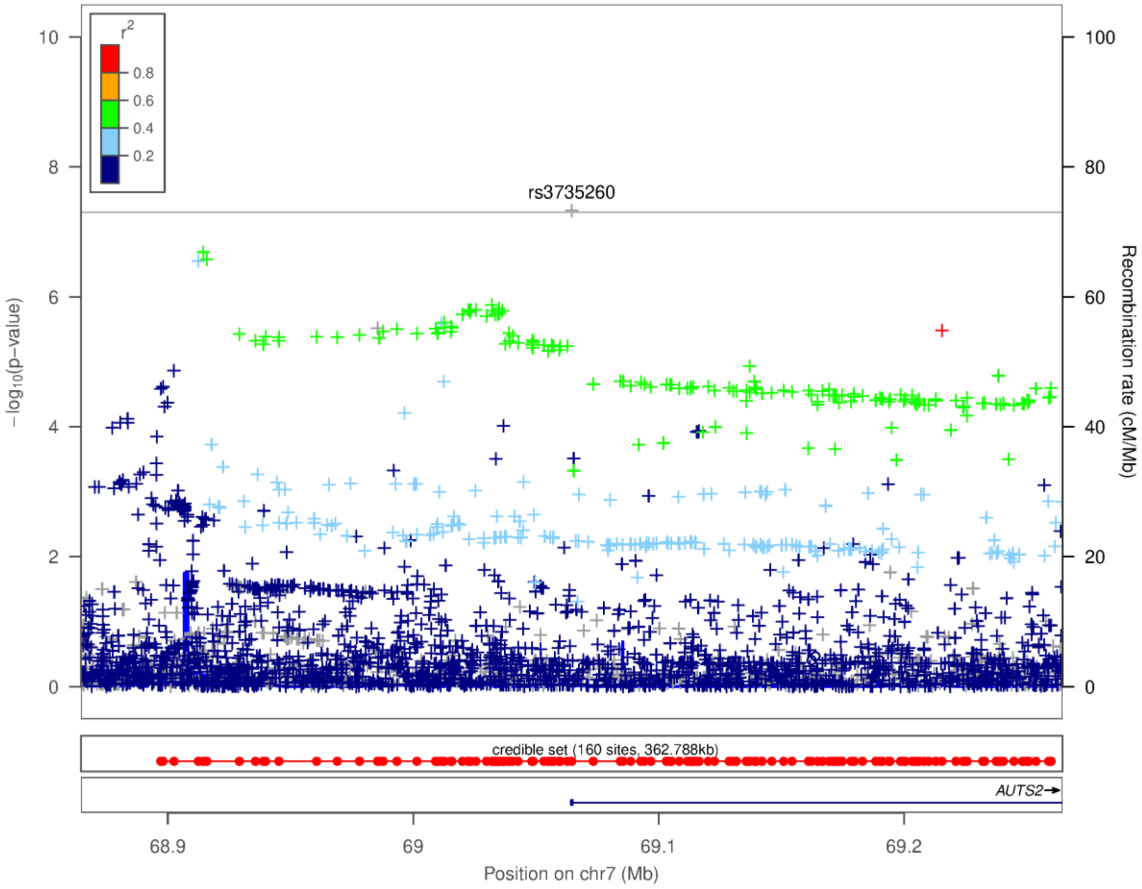

Supplementary Figure 1.xliii. Regional association plot which includes information on the credible variant set and known genes in the region for the significant association between dyslexia and chr7q11.22 rs3735260
