# Supplementary material for: Discovery of 42 genome-wide significant loci associated with dyslexia
Source: Nat Genet. 2022 Oct 20;54(11):1621–9. doi: 10.1038/s41588-022-01192-y (PMC9649434; doi:10.1038/s41588-022-01192-y)
Supplement: Supplementary file 2 — Reporting Summary [file 41588_2022_1192_MOESM2_ESM.pdf]

nature portfolio

Corresponding author(s): Michelle Luciano

Last updated by author(s): May 9, 2022

Reporting Summary

Nature Portfolio wishes to improve the reproducibility of the work that we publish. This form provides structure for consistency and transparency in reporting. For further information on Nature Portfolio policies, see our Editorial Policies and the Editorial Policy Checklist.

Statistics

For all statistical analyses, confirm that the following items are present in the figure legend, table legend, main text, or Methods section.

n/a	Confirmed	
☐	☒	The exact sample size (*n*) for each experimental group/condition, given as a discrete number and unit of measurement
☐	☒	A statement on whether measurements were taken from distinct samples or whether the same sample was measured repeatedly
☐	☒	The statistical test(s) used AND whether they are one- or two-sided<br>*Only common tests should be described solely by name; describe more complex techniques in the Methods section.*
☐	☒	A description of all covariates tested
☐	☒	A description of any assumptions or corrections, such as tests of normality and adjustment for multiple comparisons
☐	☒	A full description of the statistical parameters including central tendency (e.g. means) or other basic estimates (e.g. regression coefficient) AND variation (e.g. standard deviation) or associated estimates of uncertainty (e.g. confidence intervals)
☐	☒	For null hypothesis testing, the test statistic (e.g. *F*, *t*, *r*) with confidence intervals, effect sizes, degrees of freedom and *P* value noted<br>*Give P values as exact values whenever suitable.*
☒	☐	For Bayesian analysis, information on the choice of priors and Markov chain Monte Carlo settings
☒	☐	For hierarchical and complex designs, identification of the appropriate level for tests and full reporting of outcomes
☐	☒	Estimates of effect sizes (e.g. Cohen's *d*, Pearson's *r*), indicating how they were calculated

Our web collection on statistics for biologists contains articles on many of the points above.

Software and code

Policy information about availability of computer code

Data collection	23andMe online survey software.
Data analysis	MAGMA v1.08; FUMA v1.3.6a; LD Hub v1.9.3; LDSC (LD SCore) v1.0.1; R 4.0.0; Minimac 3 V2.0.1; PLINK 2.0<br>No custom code was written.

For manuscripts utilizing custom algorithms or software that are central to the research but not yet described in published literature, software must be made available to editors and reviewers. We strongly encourage code deposition in a community repository (e.g. GitHub). See the Nature Portfolio guidelines for submitting code & software for further information.

Data

Policy information about availability of data

All manuscripts must include a data availability statement. This statement should provide the following information, where applicable:

- Accession codes, unique identifiers, or web links for publicly available datasets
- A description of any restrictions on data availability
- For clinical datasets or third party data, please ensure that the statement adheres to our policy

The full summary statistics for each dyslexia GWAS presented in this paper will be made available through 23andMe to qualified researchers under an agreement with 23andMe that protects the privacy of the 23andMe participants. Interested investigators should email dataset-request@23andme.com and reference this paper for more information.

Field-specific reporting

Please select the one below that is the best fit for your research. If you are not sure, read the appropriate sections before making your selection.

☐ Life sciences ☒ Behavioural & social sciences ☐ Ecological, evolutionary & environmental sciences

For a reference copy of the document with all sections, see nature.com/documents/nr-reporting-summary-flat.pdf

Behavioural & social sciences study design

All studies must disclose on these points even when the disclosure is negative.

Study description	This is a quantitative study that relies on self-report data. Replication analyses draw on cognitive test data.
Research sample	The research sample are customers of 23andMe, a consumer genetics company, who have agreed to participate in research. They are slightly selected in that there is over-representation of higher socio-economic position participants. The replication samples are from the general population and some are enriched from reading difficulties.
Sampling strategy	The sample were volunteer customers of 23andMe, who consented to the use of their DNA and survey results. The largest sample size available at the time of the study were used.
Data collection	Data collection for the main analysis was online survey collection. For replication samples it was mostly in-person cognitive testing.
Timing	The main sample data include customers who consented to participate up until late 2020. The replication sample cohorts vary in data collection times with some dating back to the 90s.
Data exclusions	No exclusions were made.
Non-participation	This is not a longitudinal study so there is no sample drop-out to report.
Randomization	There was no randomization but we controlled for age and sex in the analyses.

Reporting for specific materials, systems and methods

We require information from authors about some types of materials, experimental systems and methods used in many studies. Here, indicate whether each material, system or method listed is relevant to your study. If you are not sure if a list item applies to your research, read the appropriate section before selecting a response.

Materials & experimental systems

n/a	Involved in the study	
☒	☐	Antibodies
☒	☐	Eukaryotic cell lines
☒	☐	Palaeontology and archaeology
☒	☐	Animals and other organisms
☐	☒	Human research participants
☒	☐	Clinical data
☒	☐	Dual use research of concern

Methods

n/a	Involved in the study	
☒	☐	ChIP-seq
☒	☐	Flow cytometry
☒	☐	MRI-based neuroimaging

Human research participants

Policy information about studies involving human research participants

Population characteristics	See above.
Recruitment	Participants are customers of 23andMe, so are invited to participate in general research, our study uses data from a number of online questions. There is under-representation of low socio-economic position and all participants are over 18 years.
Ethics oversight	External AAHRPP-accredited IRB, Ethical & Independent Review Services.

Note that full information on the approval of the study protocol must also be provided in the manuscript.
